# Evidence for the temporal regulation of insect segmentation by a conserved sequence of transcription factors

**DOI:** 10.1242/dev.155580

**Published:** 2018-05-23

**Authors:** Erik Clark, Andrew D. Peel

**Affiliations:** 1 Laboratory for Development and Evolution, Department of Zoology, University of Cambridge, Cambridge CB2 3EJ, UK; 2 Faculty of Biological Sciences, University of Leeds, Leeds LS2 9JT, UK

**Keywords:** *Drosophila*, Gene regulatory network, Pair-rule genes, Patterning, Segmentation, *Tribolium*

## Abstract

Long-germ insects, such as the fruit fly *Drosophila melanogaster*, pattern their segments simultaneously, whereas short-germ insects, such as the beetle *Tribolium castaneum*, pattern their segments sequentially, from anterior to posterior. Although the two modes of segmentation at first appear quite distinct, much of this difference might simply reflect developmental heterochrony. We now show here that, in both *Drosophila* and *Tribolium*, segment patterning occurs within a common framework of sequential Caudal, Dichaete and Odd-paired expression. In *Drosophila*, these transcription factors are expressed like simple timers within the blastoderm, whereas in *Tribolium* they form wavefronts that sweep from anterior to posterior across the germband. In *Drosophila*, all three are known to regulate pair-rule gene expression and influence the temporal progression of segmentation. We propose that these regulatory roles are conserved in short-germ embryos, and that therefore the changing expression profiles of these genes across insects provide a mechanistic explanation for observed differences in the timing of segmentation. In support of this hypothesis, we demonstrate that Odd-paired is essential for segmentation in *Tribolium*, contrary to previous reports.

## Abstract (precis)

**Highlighted Article:** Caudal, Dichaete and Odd-paired are likely to regulate pair-rule gene expression similarly in *Drosophila* and *Tribolium*, providing a simple mechanism for the evolutionary transition between short-germ and long-germ segmentation modes.

## INTRODUCTION

Arthropods have modular body plans composed of distinct segments serially arrayed along the anterior-posterior (AP) axis. These segments are organised and maintained by a conserved network of ‘segment-polarity’ genes, each of which is expressed in a segmentally reiterated pattern of stripes (DiNardo et al., 1994; Damen, 2002; Janssen and Budd, 2013). Intriguingly, disparate developmental strategies are used across the arthropod phylum to generate this universal segmental pattern (Peel et al., 2005). For example, early developmental stages vary dramatically between ‘long-germ’ and ‘short-germ’ insect species (Krause, 1939; Sander, 1976; Davis and Patel, 2002; Liu and Kaufman, 2005), even though the insect body plan is largely invariant.

**Fig. 1.**
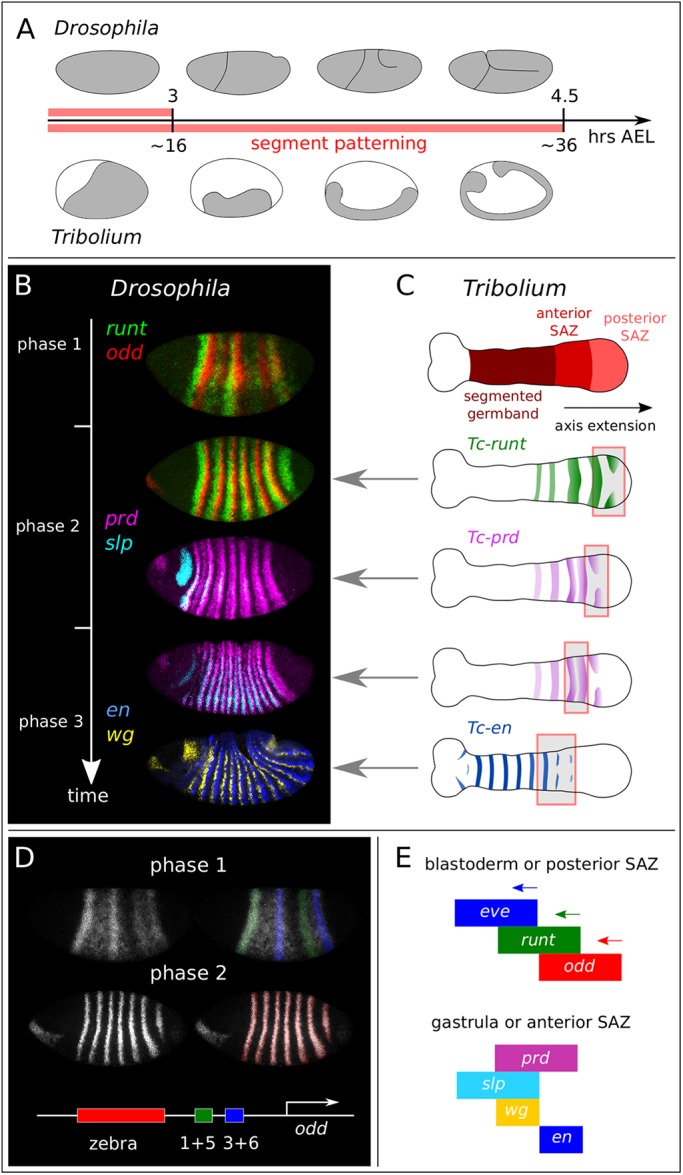
**Comparison of long-germ and short-germ segmentation.** (A) Developmental timelines for *Drosophila* and *Tribolium*. Hours until blastoderm-to-germband transition and full germband extension at 25°C are marked. Grey indicates embryonic territory. (B) Overview of *Drosophila* pair-rule patterning. Key stages of primary pair-rule, secondary pair-rule, and segment-polarity gene expression are shown in embryos of increasing age. (C) Overview of *Tribolium* pair-rule patterning, depicting equivalent gene expression in an embryo at mid germband extension. Red and grey boxes highlight similarities with the *Drosophila* patterns (left). Overview of the segment addition zone (SAZ) is at the top. (D) Enhancer organisation of *odd-skipped* (Schroeder et al., 2011), with relevant expression output highlighted above. (E) Conserved patterns of gene expression across arthropod segmentation include a dynamic sequence of primary pair-rule gene expression at early stages (top), and a specific arrangement of secondary pair-rule gene and segment-polarity gene expression domains at parasegment boundaries (bottom).

In long-germ embryos, e.g. those of the fruit fly *Drosophila melanogaster*, almost all segments are patterned during the blastoderm stage (Akam, 1987; Nasiadka et al., 2002; Fig. 1A,B). *Drosophila* uses a bespoke set of ‘stripe-specific’ enhancer elements, regulated by maternal and ‘gap’ factors, to rapidly establish a spatially periodic pattern of ‘pair-rule’ gene transcription factor expression (Schroeder et al., 2011). Pair-rule genes are expressed in patterns of seven regularly spaced stripes, reflecting a transient double-segment periodicity within the *Drosophila* embryo (Nüsslein-Volhard and Wieschaus, 1980; Hafen et al., 1984). At gastrulation, the positional information in the pair-rule pattern is used to pattern the segment-polarity genes, which are expressed in 14 stripes each (DiNardo and O'Farrell, 1987; Bouchard et al., 2000; Clark and Akam, 2016a).

In contrast, short-germ embryos, e.g. those of the beetle *Tribolium castaneum*, have retained the ancestral arthropod condition of patterning their segments sequentially from anterior to posterior over the course of embryogenesis (Patel et al., 1994; Patel, 1994; Choe et al., 2006; Choe and Brown, 2007). Short-germ embryos pattern only their anterior segments at the blastoderm stage; more posterior segments are patterned after gastrulation from a segment-addition zone (SAZ), in a process that is often coupled to embryo growth (Fig. 1A,C).

In *Tribolium*, periodic patterns do not arise from precise positioning of pair-rule stripes by gap gene orthologues (Lynch et al., 2012). Instead, the segmentation process involves sustained oscillations of pair-rule gene expression in the SAZ (Sarrazin et al., 2012; El-Sherif et al., 2012). Similar dynamic patterns of pair-rule gene expression have been reported for spiders, myriapods, crustaceans, and other short-germ insects (for example: Schönauer et al., 2016; Brena and Akam, 2013; Eriksson et al., 2013; Mito et al., 2007). These findings have drawn parallels with vertebrate somitogenesis – thought to occur via a ‘clock and wavefront’ mechanism (Cooke and Zeeman, 1976; Palmeirim et al., 1997; Oates et al., 2012), suggesting that pair-rule gene orthologues in short-germ arthropods are either components of, or entrained by, a segmentation clock (Stollewerk et al., 2003; Choe et al., 2006; Pueyo et al., 2008).

Although long-germ development is found only within holometabolous insects, the major orders within the Holometabola all contain both short-germ and long-germ species, suggesting that long-germ segmentation has evolved from a short-germ ancestral state several times independently (Davis and Patel, 2002; Jaeger, 2011). There is also at least one documented case of long-germ segmentation reverting to the short-germ state (Sucena et al., 2014). These frequent evolutionary transitions, added to the presence of numerous ‘intermediate’ modes of development, argue that the regulatory changes required to transform a short-germ embryo to a long-germ embryo are not prohibitively complex. Consistent with this, comparisons of orthologous segmentation gene expression between long-germ and short-germ arthropods have revealed striking commonalities, suggesting that the overt differences might mask an underlying conservation of mechanism, particularly for the later parts of the process (Peel et al., 2005).

First, segmentation always involves pair-rule gene orthologues expressed periodically in time and/or space. Second, there is a conserved temporal progression from the expression of ‘primary’ pair-rule genes [as defined by Schroeder et al. (2011); i.e. *hairy*, *even-skipped*, *runt*, *odd-skipped* (*odd*) and, in some species, *fushi tarazu*], to the expression of ‘secondary’ pair-rule genes [i.e. *paired* (*prd*) and *sloppy-paired* (*slp*)], and finally the expression of segment-polarity genes (e.g. *engrailed* and *wingless*). In *Drosophila*, each stage of gene expression is observed only transiently (summarised in Fig. 1B); in *Tribolium*, the whole temporal sequence can be seen throughout the period of segment addition, as a posterior-to-anterior spatial pattern along the SAZ (summarised in Fig. 1C). Finally, key aspects of the overall patterning system seem to be conserved (Fig. 1E), such as a dynamic sequence of *eve*, *runt* and *odd* expression at early stages (Choe et al., 2006; Clark, 2017), and the use of partially overlapping *prd* and *slp* domains to establish parasegment boundaries (Green and Akam, 2013).

These similarities between long-germ and short-germ segmentation could be explained if the patterning processes involved are fairly conserved, and it is mainly the timing of these processes relative to morphogenetic events that distinguishes the different modes of development (Patel et al., 1994; Akam, 1994). This possibility is supported by our recent computational modelling study, which finds that the *Drosophila* pair-rule gene network can easily be modified into a clock and wavefront-type system capable of recapitulating both long-germ and short-germ expression dynamics (Clark, 2017). The choice between these alternate macroscopic behaviours is specified, in the model, by the spatiotemporal expression patterns of extrinsic regulatory inputs that control the timing of state transitions within the pair-rule network.

Specifically, our model predicts that patterning networks in the blastoderms of long-germ insects function in the same way as those in the segment addition zones (SAZs) of short-germ insects, and that the evolution of long-germ segmentation involved heterochronic shifts in segmentation network deployment, mediated by changes to the expression patterns of key upstream regulatory factors. A similar evolutionary scenario has also been proposed recently by Zhu and colleagues (Zhu et al., 2017).

These theoretical proposals rest on two key predictions. First, there should exist broadly expressed factors that, via their influences on the segmentation network, control the temporal progression of the segmentation process. Second, the regulatory roles of these ‘timing factors’ should be widely conserved, and therefore their expression patterns should remain tightly correlated with specific phases of segmentation gene expression across all insect embryos, regardless of whether they exhibit a long-germ or short-germ mode of development.

In this manuscript, we begin to test the predictions of our model. We first establish that, in *Drosophila*, the broadly expressed segmentation genes *caudal*, *Dichaete* and *odd-paired* are each associated with specific phases of segment patterning. We then show that, as predicted, the *Tribolium* orthologues of *caudal*, *Dichaete* and *odd-paired* are expressed in the same temporal order, and preserve the same correlations with segmentation gene expression as are observed in *Drosophila*. However, whereas in *Drosophila* these factors are expressed ubiquitously throughout the trunk and thus provide only simple timers, in *Tribolium* they are expressed as retracting or advancing wavefronts, and thus could represent the primary source of spatial information within the short-germ segmentation process. Consistent with this interpretation, we find (in contrast to previous studies) that *Tc-opa* knockdown perturbs *Tribolium* segmentation. We also discover early developmental functions for *Tc-opa* in blastoderm formation and head patterning, which partially mask this segmentation role. Finally, we discuss the significance of our findings for the evolution of segmentation.

## RESULTS

### Candidates for conserved timing factors: Caudal, Dichaete and Odd-paired

We define the term ‘segmentation timing factor’ to mean a broadly expressed but temporally restricted transcriptional regulator that participates in segment patterning by modulating the expression or function of canonical (spatially patterned) segmentation genes.

Two such factors have already been identified: in *Drosophila*, the zinc-finger transcription factor Odd-paired (Opa), which triggers the onset of segment-polarity gene expression at gastrulation (Benedyk et al., 1994; Clark and Akam, 2016a), and, in *Tribolium*, the homeodomain transcription factor Caudal (Cad; Schulz et al., 1998; Macdonald and Struhl, 1986), which is thought to quantitatively tune pair-rule and gap gene expression dynamics (El-Sherif et al., 2014; Zhu et al., 2017). In *Drosophila*, Cad directly activates posterior gap genes and primary pair-rule genes (Rivera-Pomar et al., 1995; Schulz and Tautz, 1995; La Rosée et al., 1997; Häder et al., 1998; Olesnicky et al., 2006). Another prime candidate is the SOX-domain transcription factor Dichaete (Russell et al., 1996; Nambu and Nambu, 1996), which directly regulates primary pair-rule gene expression in *Drosophila* but does not noticeably affect gap gene expression (Russell et al., 1996; Ma et al., 1998; Fujioka et al., 1999; MacArthur et al., 2009; Aleksic et al., 2013). We decided to use these three genes as a first test for our evolutionary hypothesis that insect segmentation occurs within a conserved – but spatiotemporally malleable – regulatory framework that determines where and when segment patterning networks will be deployed.

### Sequential *caudal*, *Dichaete* and *odd-paired* expression correlates with the temporal progression of *Drosophila* segmentation

**Fig. 2.**
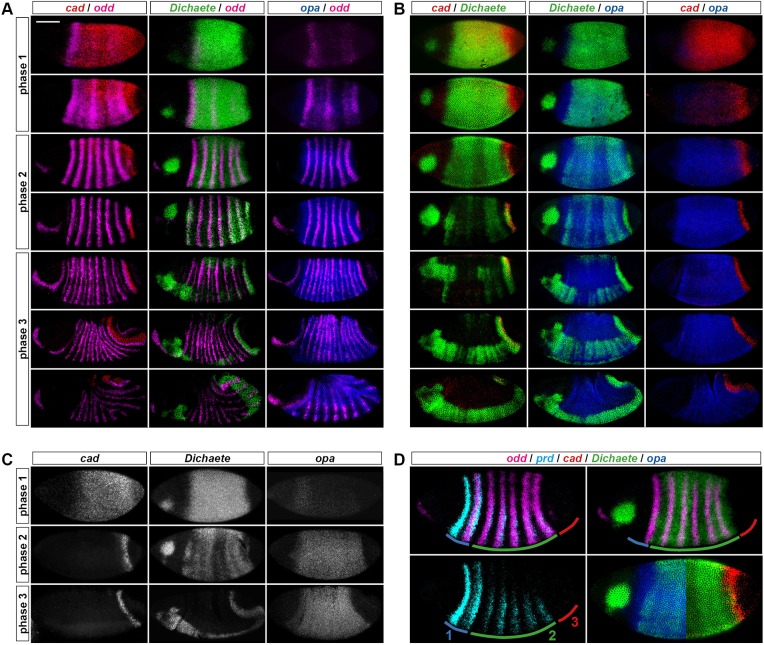
**Spatiotemporal dynamics of *cad*, *Dichaete* and *opa* during *Drosophila* segmentation.** (A) Expression relative to *odd*. (B) Expression relative to each other. (C) Summary of the overall temporal sequence. (D) Spatial correlations with segmentation timing differences along the AP axis. Embryos are all at early phase 2; annotations highlight distinct regions of pair-rule gene expression (see text); bottom-right panel combines two embryos from B. *opa*/*odd* images in rows 2-4 of A are reproduced, with permission, from Clark and Akam (2016a). Scale bar: 100 µm.

We first characterised more precisely the associations of our three candidate timing factor genes with the various phases of *Drosophila* segmentation, using the pair-rule gene *odd* as a marker (Fig. 2A). Following the staging scheme introduced by Clark and Akam (2016a), we divide pair-rule gene expression into three broad phases, spanning from early cellularisation to early germband extension (Fig. 1B). During phase 1 (early cellularisation), individual stripes of primary pair-rule gene expression are established by gap factors acting on stripe-specific enhancer elements, in most cases resulting in irregular/incomplete periodic patterns (Fig. 1D, top). During phase 2 (mid- to late-cellularisation), pair-rule factors cross-regulate through ‘zebra’ elements, resulting in regular stripes of double-segment periodicity (Fig. 1D, bottom), and the secondary pair-rule genes turn on in the trunk. During phase 3 (gastrulation onwards), the regulatory network changes and the expression patterns of some of the pair-rule genes (including *odd*) transition to single-segment periodicity.

We find that *cad* expression clears from the main trunk at the beginning of phase 2 (Fig. 2A). In contrast, *Dichaete* expression persists in the trunk throughout phase 2 (although it does become spatially modulated), clearing only at the beginning of phase 3. As described previously (Clark and Akam, 2016a), *opa* expression builds up progressively, starting from phase 1. Antibody stains indicate that the dynamics of Cad and Dichaete protein expression closely reflect their respective transcript patterns (Macdonald and Struhl, 1986; Ma et al., 1998), whereas Opa protein appears only after a significant delay (Benedyk et al., 1994), likely owing to the length of its transcription unit, which contains a 14 kb intron (FlyBase). Segmentation in *Drosophila* therefore occurs against a changing background of transcription factor expression, with phase 1 characterised by both Cad and Dichaete, phase 2 mainly by Dichaete, and phase 3 by Opa (Fig. 2C).

### *Caudal*, *Dichaete* and *odd-paired* expression correlates with segmentation timing differences along the anteroposterior axis

The expression of our candidate genes also correlates with the segmentation process across space. Although *Drosophila* segmentation is often described as ‘simultaneous’, three distinct regions along the AP axis undergo segment patterning at slightly different times (Fig. 2D; see also Surkova et al., 2008). In ‘region 1’ (near the head-trunk boundary, encompassing *odd* stripe 1 and *prd* stripes 1 and 2), both primary and secondary pair-rule genes are expressed very early, and head-specific factors play a large role in directing gene expression (Schroeder et al., 2011; Andrioli et al., 2004; Chen et al., 2012). In ‘region 2’ (the main trunk, encompassing *odd* stripes 2-6 and *prd* stripes 3-7), primary pair-rule genes turn on at phase 1, secondary pair-rules turn on during phase 2 and segment-polarity genes turn on at phase 3 (Fig. 1B). Finally, in ‘region 3’ (the tail, encompassing *odd* stripe 7 and *prd* stripe 8), the expression of specific pair-rule genes is delayed, and segment-polarity gene expression emerges only during germband extension (Kuhn et al., 2000). This region of the fate map corresponds to parasegments 14 and 15, and eventually gives rise to the terminal segments (A9 and A10) and the anal pads (Campos-Ortega and Hartenstein, 1985; Kuhn et al., 1995).

If our hypothesis is true, we would expect that spatial differences in the timing of segmentation correlate with differential expression of our three putative timing factors. Indeed, this is what we find (Fig. 2B,D).

Region 1 never expresses *cad* or *D*, but does express *opa*, because the anterior limit of the *opa* domain lies anterior to the *cad* and *D* domains. Region 2 corresponds to the early broad domain of *Dichaete* expression, which extends from just behind *odd* stripe 1 to just behind *odd* stripe 6 (the early *opa* expression domain shares the same posterior boundary). Finally, region 3 at first expresses only *cad*, because the posterior limit of the *cad* domain lies posterior to the early *D* and *opa* domains. As development proceeds, region 3 transits through the same sequence of *cad*, *D* and *opa* expression as already described for the main trunk (Fig. S1).

### *Caudal* retraction correlates with the onset of secondary pair-rule gene expression and is necessary for segment patterning

We have argued previously that the onset of Opa expression at phase 3 triggers expression pattern changes in *Drosophila* pair-rule and segment-polarity genes (Clark and Akam, 2016a). We now briefly consider the functional significance of temporally patterned Cad and Dichaete expression in *Drosophila*.

Transcription of the secondary pair-rule gene *prd* appears with a marked anterior-to-posterior and ventral-to-dorsal polarity (Kilchherr et al., 1986). Although part of the reason for this is that the early expression of *prd* stripes 1+2 is under the control of a separate Bicoid-dependent regulatory element (Gutjahr et al., 1994; Ochoa-Espinosa et al., 2005), the overall spatiotemporal pattern is still largely unexplained. Interestingly, the clearance of Cad protein from the embryo also occurs with both an anterior-to-posterior and ventral-to-dorsal polarity (Macdonald and Struhl, 1986).

**Fig. 3.**
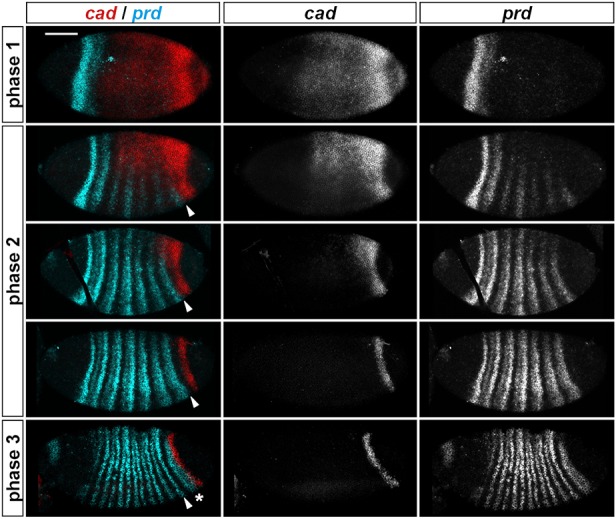
***cad* and *prd* exhibit complementary spatiotemporal dynamics.** Phase 1: the *prd* stripe 1+2 domain directly abuts *cad* expression in the trunk. Phase 2: *prd* stripes 3-7 emerge as *cad* expression retracts posteriorly and dorsally. Phase 3: *prd* stripe 8 (asterisk) emerges as the *cad* tail domain retracts posteriorly. Arrowheads mark the posterior border of *prd* stripe 7. Scale bar: 100 µm.

We therefore compared the expression of *cad* and *prd* in cycle 14 embryos and found that the emergence of the *prd* stripes is tightly spatiotemporally associated with retraction of *cad* expression (Fig. 3). This indicates that *cad* expression is specifically associated with early stages of segment patterning, before the secondary pair-rule genes turn on. Although redundancy between maternal and zygotic *cad* contributions (Macdonald and Struhl, 1986) demonstrates that segment patterning is fairly robust to quantitative variation in Cad expression, the temporal profile of Cad expression is evidently important. Cad misexpression is able to disrupt trunk segmentation when induced during the latter half of cellularisation (i.e. when the secondary pair-rule genes are expressed), but not afterwards and not before (Mlodzik et al., 1990). This finding indicates that the clearance of *cad* expression at phase 2 is crucial for normal patterning.

### Spatial regulation of *prd* is transiently compromised in *Dichaete* mutant embryos

Dichaete expression is lost from the trunk towards the end of cellularisation; therefore, any direct effects on segmentation gene expression must occur prior to gastrulation. Of the seven pair-rule genes, *hairy*, *eve*, *runt* and *ftz* have been previously examined in *Dichaete* mutant embryos, and all have been found to show well-defined, albeit irregular, seven-stripe patterns during cellularisation (Russell et al., 1996; Nambu and Nambu, 1996; Fig. S2 and S3). It is currently not clear whether these relatively subtle perturbations are caused simply by effects of Dichaete on pair-rule gene stripe-specific elements (Ma et al., 1998; Fujioka et al., 1999) or whether Dichaete is additionally acting on pair-rule gene zebra elements.

We surveyed the expression of the remaining pair-rule genes, *odd*, *prd*, and *slp*, looking for any gross misregulation (Figs S2 and S3). We found that all three genes were expressed in *Dichaete* mutant embryos, and turned on at the appropriate time, with normal DV polarity. For *odd* and *slp*, their stripes were well-defined, although the widths and spacing were abnormal. The most noticeable patterning defect was a delay in the appearance of *slp* stripe 4 (arrowheads in Fig. S2), probably a downstream effect of an unusually broad *runt* stripe 3, which patterns its anterior border (Clark and Akam, 2016a).

**Fig. 4.**
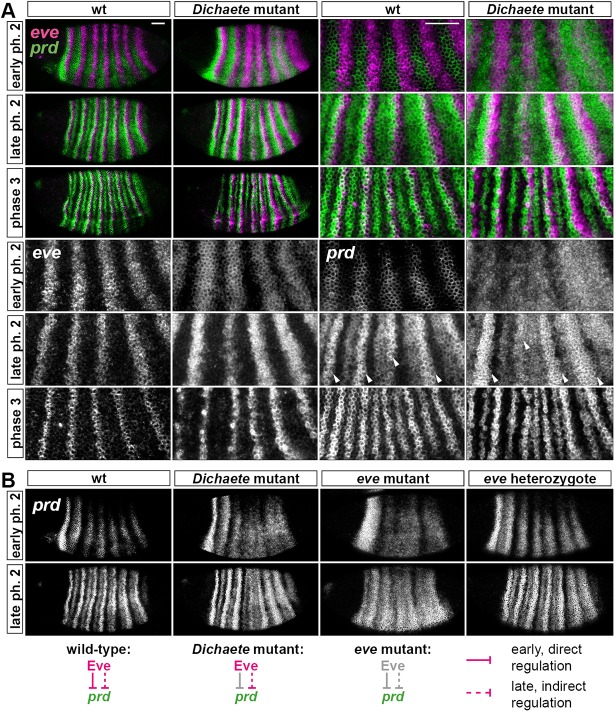
***prd* expression is perturbed in *Dichaete* mutant embryos.** (A) Ectopic *prd* expression is present at early phase 2 in *Dichaete* mutants. Enlarged views show stripes 3-6. *prd* and *eve* expression overlaps at all stages in *Dichaete* mutants, but only at later stages in wild type. Arrowheads mark ‘P’ stripes. (B) *prd* expression is aperiodic throughout phase 2 in *eve* mutants, but only at early phase 2 in *Dichaete* mutants. Scale bars: 50 µm. ph, phase.

In contrast, early *prd* patterning was severely perturbed, with stripes 3-7 fused into a broad aperiodic expression domain (Fig. 4A) (*prd* stripes 1 and 2, which lie anterior to the Dichaete domain, developed normally). *prd* trunk patterning recovered at later stages, as we saw irregular posterior (‘P’) stripe domains (Gutjahr et al., 1994) emerge at late phase 2 (arrowheads in Fig. 4A), and finally a transition to a relatively normal segment-polarity pattern at phase 3. It therefore seems that Dichaete is specifically involved in the initial phase of *prd* regulation, when basic pair-rule periodicity is established.

This phase of *prd* regulation normally consists of direct repression from Eve: the early *prd* stripes are complementary with *eve* in wild type, broaden somewhat in *eve* heterozygotes (Fig. 4B), fuse into a largely aperiodic expression domain in *eve* mutant embryos (Baumgartner and Noll, 1990) and are rapidly repressed by Eve misexpression (Manoukian and Krause, 1992). The early loss of *prd* periodicity in *Dichaete* mutant embryos resembles that in *eve* null embryos (Fig. 4B), consistent with the repression of *prd* by Eve requiring Dichaete expression. Other Eve pair-rule targets (*ftz*, *odd* and *slp*) remain out of phase with the *eve* stripes in *Dichaete* mutant embryos (Fig. S3), rather than being ectopically expressed as in *eve* mutant embryos (see Clark, 2017). This suggests that any functional interaction between Dichaete and Eve is specific to *prd* regulation.

The effect of Dichaete on *prd* expression presumably involves the *prd* zebra element and therefore implicates Dichaete as an extrinsic regulator of the pair-rule network analogous to Opa. As with Cad, heat-shock-mediated misexpression of Dichaete during cellularisation causes severe segmentation defects (Russell et al., 1996), indicating that an appropriate temporal profile of Dichaete expression is crucial for patterning. We can therefore conclude that Cad, Dichaete and Opa all temporally regulate the segmentation process in *Drosophila*, although the roles of Cad and Dichaete still need to be fully elucidated.

### Staggered wavefronts of *Tc-caudal*, *Tc-Dichaete* and *Tc-odd-paired* preserve the sequence of timing factor expression in the *Tribolium* SAZ

If long-germ segmentation does indeed represent a heterochronic shift in the deployment of a conserved patterning machinery, correlations between the expression patterns of our three putative timing factors and those of the canonical segmentation genes should be preserved in short-germ insects. We therefore examined the expression of their orthologues, *Tc-cad*, *Tc-Dichaete* and *Tc-opa*, in the short-germ beetle *Tribolium castaneum*. The expression patterns of all three have been described previously (Schulz et al., 1998; Oberhofer et al., 2014; Choe et al., 2017; Zhu et al., 2017), but only for a few stages and not in combination, providing only a limited understanding of their spatiotemporal dynamics over the course of segmentation.

**Fig. 5.**
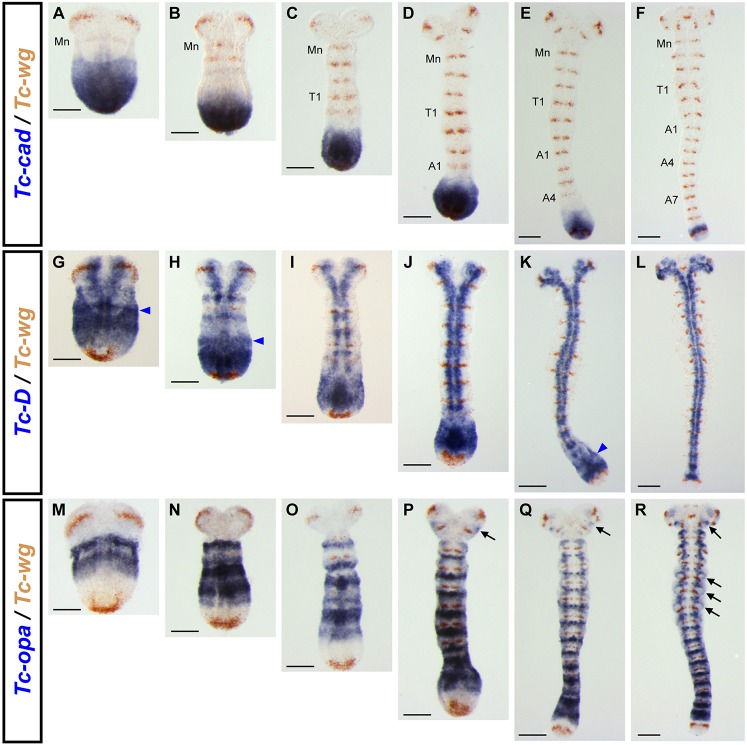
**Spatiotemporal dynamics of *Tc-cad*, *Tc-Dichaete* and *Tc-opa* during *Tribolium* segmentation.** Germband age increases from left to right; columns are stage matched by *Tc-wg* expression (Mn, mandibular; T1, prothoracic; A1/4/7, 1st/4th/7th abdominal). Blue arrowheads in G,H,K indicate a *Tc-Dichaete* stripe anterior to the strong posterior expression domain. Black arrows in P-R mark *Tc-opa* expression in the antennae and at the bases of the thoracic legs. Scale bars: 100 µm.

Fig. 5 shows staged expression of *Tc-cad*, *Tc-Dichaete* and *Tc-opa*, all relative to a common marker, *Tc-wg*, over the course of germband extension. A more extensive set of stages is shown in Fig. S4, and direct comparisons between *Tc-cad*/*Tc-Dichaete*, *Tc-cad*/*Tc-opa* and *Tc-Dichaete*/*Tc-opa* are shown in Fig. S5.

*Tc-cad* is continuously expressed in the SAZ, resulting in a persistent posterior domain that gradually shrinks over time as the germband elongates (a process that depends on convergent extension cell movements; Nakamoto et al., 2015; Benton et al., 2016). *Tc-cad* therefore turns off in presegmental tissues as they emerge from the anterior of the SAZ, well before the *Tc-wg* stripes turn on. Faint pair-rule stripes of *Tc-cad* are sometimes seen in the anterior SAZ, paralleling similar stripes seen in *Drosophila* at early phase 3 (Fig. S6A,B).

*Tc-Dichaete* is also broadly expressed within the SAZ, but is excluded from the most posterior tissue, turning on slightly anterior to the terminal (circum-proctodaeal) *Tc-wg* domain. The SAZ expression of *Tc-Dichaete* extends slightly further to the anterior than that of *Tc-cad*, turning off just before the *Tc-wg* stripes turn on. *Tc-Dichaete* expression anterior to the *Tc-cad* domain tends to be at lower levels and/or periodically modulated; in some embryos, we observe a separate stripe of *Tc-Dichaete* expression, anterior to the broad SAZ domain (Fig. 5G,H,K). *Tc-Dichaete* expression later transitions into persistent expression within the neuroectoderm, with expression now absent from the more lateral ectodermal regions. This same general sequence, from strong uniform expression, to weaker and periodically modulated expression, to neuroectodermal expression, is also observed in *Drosophila* development (Fig. 2A,B).

Finally, *Tc-opa* is absent from the posterior half of the SAZ, but is expressed in a broad posterior domain starting in the anterior SAZ and extending anteriorly to surround nascent *Tc-wg* stripes. The intensity of expression in this domain is spatially modulated, with *Tc-opa* expression transitioning more anteriorly into relatively persistent segmental stripes, which cover the central third of each parasegment (Fig. S6D). A transition to segmental expression also occurs in *Drosophila*, during germband extension (Fig. S6C).

Importantly, the overall pattern of expression is consistent throughout germband elongation: the posterior expression domains of each of the three genes retain the same relationship to the gross morphology of the embryo, and to each other, at each timepoint. This means that during germband elongation, each cell within the SAZ will at some point experience a temporal progression through the three transcription factors, similar to that experienced by cells within the *Drosophila* trunk over the course of cellularisation and gastrulation (compare Fig. 2). For most of the cells that contribute to the *Tribolium* trunk, this sequence is likely to start with Cad+Dichaete, transit through Dichaete+Opa and end with Opa alone.

### Correlations with segmentation gene expression are broadly conserved in *Tribolium*

**Fig. 6.**
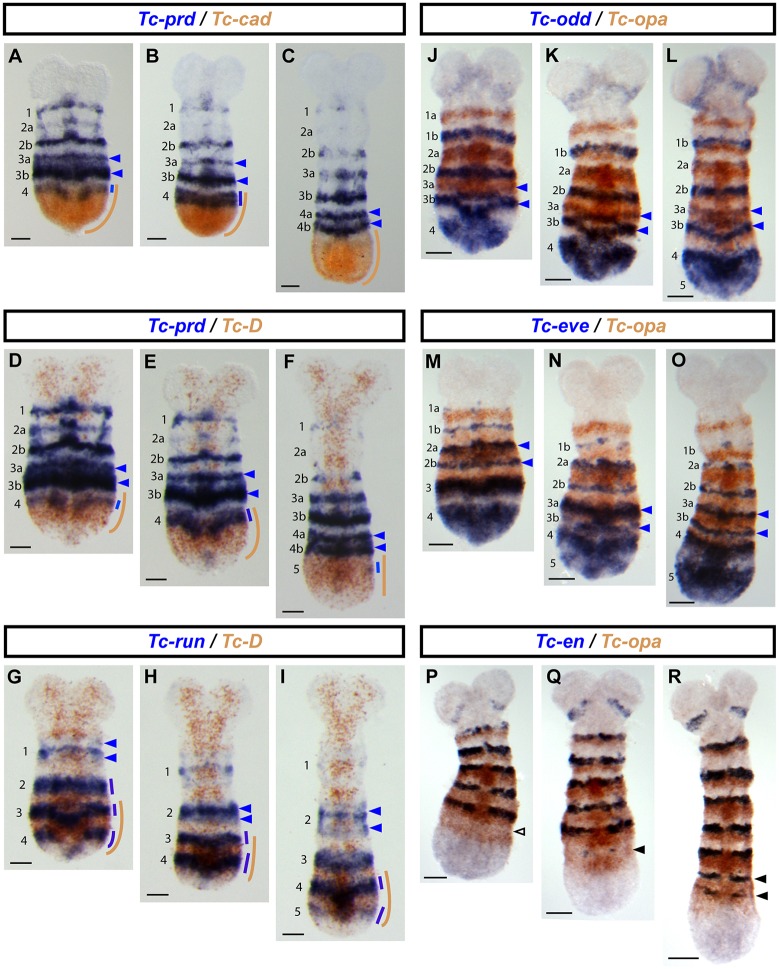
**Expression of *Tc-cad*, *Tc-Dichaete* and *Tc-opa* relative to selected segmentation genes.** (A-F) The 4th *Tc-prd* primary pair-rule stripe forms at the anterior of the *Tc-cad* (A,B) and *Tc-Dichaete* (D,E) domains, and splits to form segmental stripes (4a and 4b) anteriorly to these domains (C,F). (G-I) The 4th and 5th *Tc-run* primary pair-rule stripes form at the posterior of the *Tc-Dichaete* domain, while the 2nd primary stripe splits anteriorly to the Tc-Dichaete domain. (J-O) The 4th and 5th *Tc-odd*/*Tc-eve* primary pair-rule stripes form posteriorly to the *Tc-opa* domain, while the 3rd *Tc-odd*/*Tc-eve* primary pair-rule stripes resolve into segmental stripes (3a and 3b) within the *Tc-opa* domain. (P-R) The T3 and A1 *Tc-en* stripes form within the *Tc-opa* domain. Nascent segmental stripes (solid black arrowheads) emerge from a region where *Tc-opa* expression is already clearing (empty black arrowhead). Blue arrowheads in A-O mark resolving or recently resolved segmental stripes; colour-coded lines in A-I indicate the extent of expression domains. Scale bars: 50 µm.

We also compared the expression domains of *Tc-cad*, *Tc-Dichaete* and *Tc-opa* with the expression patterns of key *Tribolium* segmentation genes (Fig. 6; and more extensive developmental series in Figs S7-S10). As expected, we found that the expression of each factor correlates with specific phases of segmentation gene expression, and that these correlations are very similar to those observed in *Drosophila*.

In *Drosophila*, we found that the onset of *prd* expression correlated with the retraction of *cad* expression (Fig. 3), and that the early, pair-rule phase of *prd* expression involved regulation by Dichaete (Fig. 4); in *Tribolium*, *Tc-prd* turns on near the anterior limit of the *Tc-cad* domain (Fig. 6A-C; Fig. S7), with the pair-rule phase of *Tc-prd* expression falling within the *Tc-Dichaete* domain, and stripe splitting occurring anterior to it (Fig. 6D-F; Fig. S8). In *Drosophila*, we found that the primary pair-rule genes turn on in the context of strong *Dichaete* expression (Fig. 2); in *Tribolium*, the *Tc-runt* pair-rule stripes turn on at the very posterior of the *Tc-Dichaete* domain, emanating from two lateral spots either side of the posterior *Tc-wg* domain (Fig. 6G-I; Fig. S9). The *Tc-eve* and *Tc-odd* stripes also emerge with similar dynamics (Fig. 6J-O; Fig. S10F-O; Sarrazin et al., 2012). Finally, in *Drosophila*, Opa is required for the frequency doubling of pair-rule gene expression and the activation of segment-polarity gene expression (Clark and Akam, 2016a; Benedyk et al., 1994); in *Tribolium*, frequency doubling of all the pair-rule genes examined occurs within the *Tc-opa* domain, and the stripes of the segment-polarity genes *Tc-en* and *Tc-wg* emerge within it, as well (Fig. 6J-R; Fig. S10A-E).

The temporal progression of the segmentation process therefore seems to be remarkably similar in both species, albeit in a different spatiotemporal deployment: primary pair-rule genes are expressed dynamically in the context of Cad and Dichaete expression, secondary pair-rule genes turn on as Cad turns off, and frequency doubling and segment-polarity activation occur in the context of Opa expression.

### Parental RNAi for *Tc-opa* yields empty eggs and head-patterning defects

The expression patterns of *Tc-cad*, *Tc-Dichaete*, and *Tc-opa* are consistent with these three genes forming part of a conserved temporal framework that regulates insect segmentation. However, this hypothesis is not supported by existing *Tribolium* RNAi studies, which conclude that *Tc-opa* is not involved in segmentation (Choe et al., 2006, 2017). In contrast, the iBeetle RNAi screen (Dönitz et al., 2015) does show severe pair-rule-like segmentation defects for *Tc-opa* that are consistent with our proposal. As a first-pass screen, results from iBeetle need to be independently verified. We therefore performed our own *Tc-opa* RNAi experiments to clarify the situation (for full results, see Tables S1-S3).

**Fig. 7.**
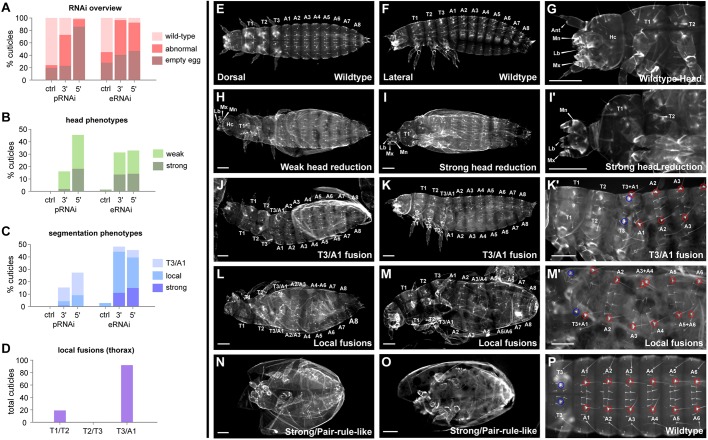
***Tc-opa* RNAi reveals head and segmentation roles.** (A) Summary results for pRNAi and eRNAi, compared with sham-injected controls. (B,C) Quantification of head and segmentation phenotypes following RNAi. (pRNAi data are from a different experiment from that shown in A, see Table S1.) (D) Counts of RNAi-induced local segment fusions within the thorax (all experiments). (E-G,P) Wild-type larval cuticles. (H-I′) Representative larval cuticles with RNAi-induced head phenotypes. (J-O) Representative larval cuticles with RNAi-induced segmentation phenotypes. Blue (thoracic) and red (abdominal) circles in K′,M′,P highlight the relative position of homologous bristles. Scale bars: 100 µm. See Tables S1-S3 and Figs S11, S12 and S14 for more details.

We first carried out parental RNAi (pRNAi) experiments using two non-overlapping fragments, corresponding to the 5′ and 3′ exons of the *Tc-opa* gene. The 5′ dsRNA injections resulted mainly in empty eggs (257/300; 86%) and very few wild-type cuticles (6/300; 2%). In contrast, the 3′ dsRNA injections resulted in a much smaller proportion of empty eggs (68/300; 23%) and many more wild-type cuticles (82/300; 38%), consistent with a weaker knockdown efficacy (Fig. 7A).

**Fig. 8.**
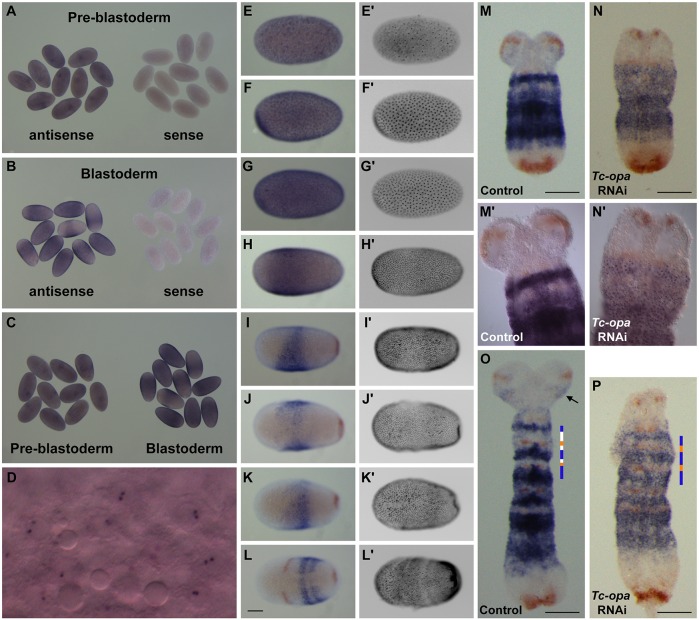
***Tc-opa* expression in wild-type blastoderms and *Tc-opa* RNAi germbands.** (A-C) *Tc-opa* mRNA in early embryos. Eggs in each panel were imaged simultaneously, all panels use the same microscope/camera settings. (D) High-resolution image of the egg in F, revealing nascent nuclear *Tc-opa* transcripts (‘nuclear dots’) in energids surfacing to form the blastoderm. (E-L′) Blastoderm eggs of increasing age stained for *Tc-opa* (E-L, blue) and *Tc-wg* (I-L, red), staged using DAPI staining (E′-L′). (M-P) Germband stage embryos from *Tc-opa* RNAi females stained for *Tc-opa*, compared with controls from sham-injected females (*Tc-wg*, red, used for stage matching). Note reduced head lobes and punctate *Tc-opa* expression (N′), reflecting reduced transcript levels in the cytoplasm and strong nuclear dots. Black arrow in O indicates antennal *Tc-opa* and *Tc-wg* domains, missing from P. Coloured bars in O,P highlight an altered segment-polarity pattern in P. Scale bars: 100 µm.

Importantly, cuticles showed similar minor segmentation defects in both pRNAi experiments, in particular a fusion between T3 and A1 (Fig. 7J-K′; Fig. S11). However, severe segmentation defects were seen only in the 3′ pRNAi and accounted for less than 1% of the cuticles. Phenotypic cuticles showed a range of other defects, which were very similar in nature across the 5′ and 3′ experiments, relating to the antennae (twisted) and legs (often one bifurcated T2 appendage) (Fig. S12). Larvae with these relatively minor phenotypes often hatched, but exhibited abnormal movement and died before the first moult. Some cuticles exhibited more dramatic head-patterning defects (loss of labrum, antennae and the head capsule; Fig. 8H-I′). These phenotypes were more common (as a percentage of total cuticles) and more severe (i.e. more with complete loss of head capsule) in the 5′ pRNAi experiment (Fig. 7B), consistent with the stronger effect 5′ dsRNA had on embryo viability relative to 3′ dsRNA.

### *Tc-opa* is expressed broadly at blastodermal and pre-blastodermal stages

With respect to segmentation phenotypes, our pRNAi experiments yielded results that were not too dissimilar to those of Choe and colleagues. However, we were intrigued by the frequent head phenotypes in both our 3′ and 5′ pRNAi data and the iBeetle screen. We therefore analysed *Tc-opa* expression in blastoderm eggs and discovered that strong *Tc-opa* expression prior to the appearance of the mandibular stripe had previously been overlooked.

We found that *Tc-opa* was both maternally provided (ubiquitous mRNA at pre-blastoderm stages, Fig. 8A) and zygotically expressed at high levels in the earliest blastoderm stages (Fig. 8B-F), at first ubiquitously (Fig. 8G), but resolving towards the end of the uniform blastoderm stage into a wedge-shaped region covering the future anterior head anlagen (Fig. 8H-K). These early expression domains might therefore explain the high levels of embryonic lethality and/or head phenotypes resulting from our pRNAi injections. The RNAi defects associated with appendages (i.e. antennae and legs) also correlated with specific *Tc-opa* expression domains (Fig. 5P-R), such that overall there was a tight association between the cuticle defects we observed following RNAi and the numerous domains of *Tc-opa* expression.

### Embryonic RNAi for *Tc-opa* reveals an important role in segmentation

We hypothesized that a crucial blastoderm role for Opa might arrest development at early stages in eggs where *Tc-opa* is strongly knocked down, precluding the appearance of severe segmentation phenotypes in our pRNAi experiments and in those of Choe and colleagues. Consistent with this idea, fixations of 48 h (30°C) egg collections from 5′ pRNAi females revealed very few germband stage embryos (7/311; 2%) compared with controls (459/669; 67%). DAPI staining of the germband-less eggs revealed that very few had reached the blastoderm stage (1/50; 2%) but many had commenced nuclear divisions (34/50; 68%), indicating that embryonic development was starting, but usually stalling before the blastoderm stage (Fig. S13).

To bypass these early roles of *Tc-opa* in embryogenesis, we decided to perform embryonic RNAi (eRNAi), using the same 5′ and 3′ dsRNA fragments. Egg injections of *Tc-opa* dsRNA were carried out at pre-blastoderm to early blastoderm stage (2-4 h AEL as measured at 30°C), alongside control injections of buffer. In agreement with our supposition that early *Tc-opa* expression is necessary for development, the prevalence of empty eggs resulting from these injections (3′: 80/198; 40%, and 5′: 117/252; 46%) was relatively low compared with 5′ pRNAi (257/300; 86%), and not that much higher than observed in control embryonic injections (55/198; 28%) (Fig. 7A).

Strikingly, the proportion and severity of segmentation phenotypes increased dramatically with eRNAi compared with pRNAi (Fig. 7C). We observed a phenotypic series in segmentation defects, ranging from local segment fusions (Fig. 7L-M′), as seen in the pRNAi experiments, through to canonical pair-rule phenotypes (Fig. 7N,O), as reported in the iBeetle screen, and finally compacted balls of cuticle or cuticle fragments, sometimes with only a hindgut remaining (Fig. S14). In the thorax, the fusions always involved the loss of odd-numbered segment boundaries (Fig. 7D), just as seen in *opa* mutant cuticles in *Drosophila* (Jürgens et al., 1984; Benedyk et al., 1994). Fusions in the abdomen were typically more extensive, involving both odd-numbered and even-numbered boundaries. The 5′ and 3′ eRNAi phenotypes were very similar in type and frequency, ruling out off-target RNAi effects, and their relative absence from injection controls and similarity to pRNAi phenotypes argues against injection artefacts (Figs. S11 and 12; Tables S1-S3). Taken together, these results indicate that *opa* is indeed (in addition to many other roles) a segmentation gene in *Tribolium*, and that its segment patterning role is likely at least partially conserved between long-germ and short-germ insects.

### Surviving *Tc-opa* pRNAi germbands exhibit a range of defects correlated with cuticle phenotypes

The appearance of strong head phenotypes but not strong segmentation phenotypes in our pRNAi experiments suggests that the head patterning function of *Tc-opa* is more sensitive to RNAi than both the blastoderm and segmentation functions. Indeed, when we examined pRNAi germbands (Fig. 8M-P), we found several with much reduced head lobes (presumably corresponding to the head phenotypes observed in the cuticles) and these had essentially normal segmental *Tc-wg* expression. We also observed a loss of antennal *Tc-wg* expression and an asymmetric ectopic stripe of *Tc-wg* expression within the second thoracic segment, correlating with antennal abnormalities and T2 leg bifurcations, respectively (Fig. S12J-O).

In these pRNAi embryos, cytoplasmic *Tc-opa* expression was largely absent, indicating that the RNAi had at least been partially effective. However, the embryos exhibited very strong nuclear dots (Fig. 8N′), indicating that *Tc-opa* was being transcribed at high levels, and might be hard to knock down completely using pRNAi. In addition, although the *Tc-wg* stripes in these embryos indicated successful segment boundary formation, the germbands were shorter and fatter than in wild type and the pattern of *Tc-opa* expression was abnormal. These observations indicate that subtle AP patterning defects (such as convergent-extension problems and segment-polarity abnormalities) occur even in partial knockdowns, perhaps explaining the local segment fusions we observed in pRNAi cuticles.

## DISCUSSION

**Fig. 9.**
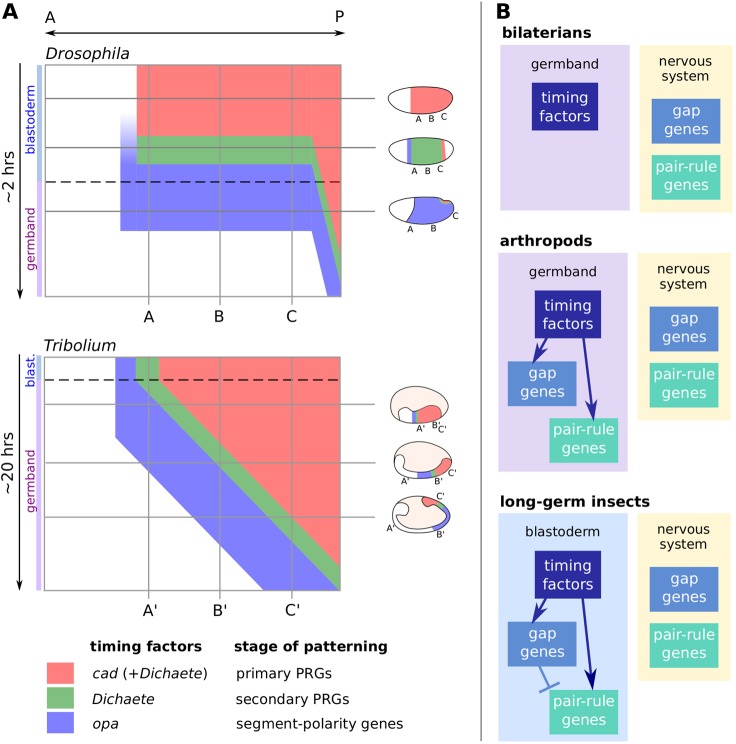
**A conserved regulatory framework for arthropod segmentation.** (A) Schematic comparison of timing factor expression during *Drosophila* versus *Tribolium* segmentation. Kymographs depict expression along the ectodermal AP axis over time. Dotted lines mark blastoderm-to-germband transition. Neural and segment-polarity expression domains are not drawn. (B) Proposed scenario for the evolution of arthropod segmentation: ancestrally, timing factors regulated AP axis extension and ‘segmentation’ genes had neural functions (top); the evolution of short-germ segmentation involved segmentation genes coming under the spatiotemporal regulation of the timing factors within the germband (middle); the evolution of long-germ segmentation involved the timing factors being expressed earlier in development and the pair-rule genes being spatially regulated by the gap genes (bottom).

We have found that segment patterning in both *Drosophila* and *Tribolium* occurs within a conserved framework of sequential Caudal, Dichaete and Odd-paired expression. In the case of Opa, we also have evidence for conserved function. However, although the sequence itself is conserved between the two insects, its spatiotemporal deployment across the embryo is divergent (Fig. 9A). In *Drosophila*, the factors are expressed ubiquitously within the main trunk, and each turns on or off almost simultaneously, correlating with the temporal progression of a near simultaneous segmentation process. In *Tribolium*, their expression domains are staggered in space, with developmentally more advanced anterior regions always subjected to a ‘later’ regulatory signature than more-posterior tissue. These expression domains retract over the course of germband extension, correlating with the temporal progression of a sequential segmentation process built around a segmentation clock.

### Orchestration of complex developmental processes by extrinsic timing factors

Pair-rule patterning involves several distinct phases of gene expression, each requiring specific regulatory logic (Clark and Akam, 2016a). We propose that, in both long-germ and short-germ species, the whole process is orchestrated by a series of key regulators, expressed sequentially over time, three of which we have focused on in this manuscript. By rewiring the regulatory connections between other genes, factors such as Dichaete and Opa allow a small set of pair-rule factors to carry out multiple different roles, each specific to a particular spatiotemporal regulatory context. This kind of control logic makes for a flexible, modular regulatory network, and may therefore turn out to be a hallmark of other complex patterning systems.

Having highlighted the significance of these ‘timing factors’ in this manuscript, the next steps will be to investigate their precise regulatory roles and modes of action. It will be interesting to dissect how genetic interactions with pair-rule factors are implemented at the molecular level. Dichaete is known to act both as a repressive co-factor (Zhao and Skeath, 2002; Zhao et al., 2007) and as a transcriptional activator (Aleksic et al., 2013); therefore, a number of different mechanisms are plausible. The Odd-paired protein is also likely to possess both these kinds of regulatory activities (Ali et al., 2012).

### Conserved temporal regulation of insect segmentation?

Given the phylogenetic distance between beetles and flies (separated by at least 300 million years, Wolfe et al., 2016), we expect that the similarities we see between *Drosophila* and *Tribolium* segmentation are likely to hold true for other insects, and perhaps for many non-insect arthropods as well. We propose that these similarities, which argue for the homology of long-germ and short-germ segmentation processes, result from conserved roles of Cad, Dichaete and Opa in the temporal regulation of pair-rule and segment-polarity gene expression during segment patterning. This hypothesis can be tested by detailed comparative studies in various arthropod model organisms.

Above, we have provided evidence that a segmentation role for Opa is conserved between *Drosophila* and *Tribolium*; clear segmentation phenotypes have also been found for Cad in *Nasonia* (Olesnicky et al., 2006), and for Dichaete in *Bombyx* (Nakao, 2017). However, as our *Tc-opa* experiments reveal, functional manipulations in short-germ insects will need to be designed carefully in order to bypass the early roles of these pleiotropic genes. For example, *cad* knockdowns cause severe axis truncations in many arthropods (Copf et al., 2004), whereas *Dichaete* knockdown in *Tribolium* yields mainly empty eggs (Oberhofer et al., 2014).

### Opa is a key developmental transcription factor in *Tribolium*

It was previously thought that *Tc-opa* was not required for segmentation (Choe et al., 2006,  2017), and that the segmentation role of Opa may have been recently acquired, in the lineage leading to *Drosophila* (Choe and Brown, 2007). However, our analysis reveals that *Tc-opa* is indeed a segmentation gene, and also has other important roles, including head patterning and blastoderm formation. Given that a similar developmental profile of *opa* expression is seen in the millipede *Glomeris* (Janssen et al., 2011), and even in the onychophoran *Euperipatoides* (Janssen and Budd, 2013), we think that the segmentation role of Opa may actually be ancient.

Head phenotypes following *Tc-opa* RNAi were unexpected, but both the blastoderm expression pattern and cuticle phenotypes we observe are strikingly similar to those reported for *Tc-otd* and *Tc-ems* (*Tribolium* orthologues of the *Drosophila* head ‘gap’ genes *orthodenticle* and *empty spiracles*; Schinko et al., 2008), suggesting that the three genes function together in a gene network that controls early head patterning. This function of *Tc-opa* might be homologous to the head patterning role for Opa discovered in the spider *Parasteatoda* (Kanayama et al., 2011), where it interacts with both Otd and Hedgehog (Hh) expression. Opa/Zic is known to modulate Hh signalling (Koyabu et al., 2001; Chan et al., 2011; Quinn et al., 2012), and a role for Hh in head patterning appears to be conserved across arthropods, including *Tribolium* (Farzana and Brown, 2008; Hunnekuhl and Akam, 2017).

Finally, Opa/Zic is also known to modulate Wnt signalling (Murgan et al., 2015; Pourebrahim et al., 2011). In chordates, Zic expression tends to overlap with sites of Hh and/or Wnt signalling, suggesting that one of its key roles in development is to ensure cells respond appropriately to these signals (Fujimi et al., 2012; Sanek et al., 2009; Chervenak et al., 2013; Houtmeyers et al., 2013). The expression domains of *Tc-opa* that we observe in *Tribolium* (e.g. in the head, in the SAZ and between parasegment boundaries) accord well with this idea.

### The evolution of arthropod segmentation

Similar embryonic expression patterns of Cad, Dichaete and Opa orthologues are observed in other bilaterian clades, including vertebrates. Cdx genes are expressed in the posterior of vertebrate embryos, where they play crucial roles in axial extension and Hox gene regulation (van Rooijen et al., 2012; Neijts et al., 2016). Sox2 (a Dichaete orthologue) has conserved expression in the nervous system, but is also expressed in a posterior domain, where it is a key determinant of neuromesodermal progenitor (posterior stem cell) fate (Wood and Episkopou, 1999; Wymeersch et al., 2016). Finally, Zic2 and Zic3 (Opa orthologues) are expressed in presomitic mesoderm and nascent somites, and have been functionally implicated in somitogenesis and convergent extension (Inoue et al., 2007; Cast et al., 2012). All three factors thus have important functions in posterior elongation, roles that may well be conserved across Bilateria (Copf et al., 2004).

In *Tribolium*, we think that all three factors may be integrated into an ancient gene regulatory network downstream of posterior Wnt signalling, which generates their sequential expression and helps regulate posterior proliferation and/or differentiation (McGregor et al., 2009; Oberhofer et al., 2014; Williams and Nagy, 2016). The mutually exclusive patterns of *Tc-wg* and *Tc-Dichaete* in the posterior germband are particularly suggestive: Wnt signalling and Sox gene expression are known to interact in many developmental contexts (Kormish et al., 2010) and these interactions may form parts of temporal cascades (Agathocleous et al., 2009).

We therefore suggest the following outline as a plausible scenario for the evolution of arthropod segmentation (Fig. 9B).

1. In non-segmented bilaterian ancestors of the arthropods, Cad, Dichaete and Opa were expressed broadly similarly to how they are expressed in *Tribolium* today, mediating conserved roles in posterior elongation, while gap and pair-rule genes may have had functions in the nervous system (Isshiki et al., 2001; Doe et al., 1988; Mondal et al., 2007; Shimojo et al., 2008; Janssen and Budd, 2013).
2. At some point, segmentation genes came under the regulatory control of these factors, which provided a pre-existing source of spatiotemporal information in the developing embryo. Pair-rule genes began oscillating in the posterior, perhaps under the control of Cad (El-Sherif et al., 2014; Schönauer et al., 2016) and/or Dichaete, while the posteriorly retracting expression boundaries of the timing factors provided smooth wavefronts that effectively translated these oscillations into periodic patterning of the AP axis, analogous to the roles of the opposing retinoic acid and FGF gradients in vertebrate somitogenesis (Oates et al., 2012).
3. Much later, in certain lineages of holometabolous insects, the transition to long-germ segmentation occurred. This would have involved two main modifications of the short-germ segmentation process: (i) changes to the expression of the timing factors, away from the situation seen in *Tribolium*, and towards the situation seen in *Drosophila*, causing a heterochronic shift in the deployment of the segmentation machinery from SAZ to blastoderm; and (ii) recruitment of gap genes to pattern pair-rule stripes, via the ad hoc evolution of stripe-specific elements (Peel and Akam, 2003; Rosenberg et al., 2014).

The appeal of this model is that the co-option of existing developmental features at each stage reduces the number of regulatory changes required to evolve *de novo*, facilitating the evolutionary process. In this scenario, arthropod segmentation would not be homologous to segmentation in other phyla, but would probably have been fashioned from common parts (Chipman, 2010; Graham et al., 2014).

## MATERIALS AND METHODS

### Drosophila melanogaster

Embryo collections were carried out at 25°C. The *Drosophila* mutants used were *D*^r72^ (a gift from Steve Russell, University of Cambridge, UK) and *eve*^3^ (a gift from Bénédicte Sanson, University of Cambridge, UK). Wild-type flies were Oregon-R. In order to distinguish homozygous mutant embryos, mutant alleles were balanced over *CyO hb::lacZ* (Bloomington stock number 6650). DIG-labelled and FITC-labelled riboprobes were generated using full-length cDNAs from the *Drosophila* gene collection (Stapleton et al., 2002), obtained from the *Drosophila* Genomics Resources Centre. The clones used were LD29596 (*cad*), LD16125 (*opa*), RE40955 (*hairy*), MIP30861 (*eve*), IP01266 (*runt*), GH22686 (*ftz*), RE48009 (*odd*), GH04704 (*prd*), LD30441 (*slp*) and F107617 (*en*). The cDNA for *Dichaete* was a gift from Steve Russell and the cDNA for *lacZ* was a gift from Nan Hu (University of Cambridge, UK).

Double fluorescent *in situ* hybridisation was carried out as described previously (Clark and Akam, 2016a). Images were acquired using a Leica SP5 confocal microscope. Contrast and brightness adjustments of images were carried out using Fiji (Schindelin et al., 2012; Schneider et al., 2012). Some of the wild-type images were taken from a previously published dataset (Clark and Akam, 2016b).

### Tribolium castaneum

#### Whole-mount *in situ* hybridisation

*Tribolium castaneum* eggs (San Bernardino strain) were collected on organic plain white flour (Doves Farm Foods, Hungerford, UK) at 30°C over a period of 48 h. Alkaline phosphatase *in situ* hybridisation on whole-mount embryos were carried out as previously described (Schinko et al., 2009). RNA probes were DIG labelled (all genes) and in most cases also FITC labelled (*Tc-Dichaete*, *Tc-opa*, *Tc-prd*, *Tc-wg* and *Tc-en*) and prepared according to Kosman et al. (2004), using gene fragments amplified from *Tribolium castaneum* genomic DNA (for *Tc-cad*, *Tc-eve*, *Tc-odd* and *Tc-run*) or cDNA (for *Tc-Dichaete*, *Tc-opa*, *Tc-prd*, *Tc-wg* and *Tc-en*) and cloned into the pGEM-T Easy Vector (Promega).

The generation of DIG-labelled probes against *Tc-cad* has been previously described by Peel and Averof (2010), and the generation of DIG-labelled probes against *Tc-eve* and *Tc-odd* has been previously described by Sarrazin et al. (2012). The remaining gene fragments were amplified using the following primers: *Tc-run*, 5′-CAACAAGAGCCTGCCCATC-3′ and 5′-TACGGCCTCCACACACTTT-3′ (amplifies a 3158 bp fragment); *Tc-Dichaete* (TC013163), 5′-TAACAACCGACACCCAACAG-3′ and 5′-TTGACGACCACAGCGATAATAA-3′ (921 bp fragment); *Tc-opa* (TC010234), 5′-CCCAAGAATGGCCTACTGC-3′ and 5′-TTGAAGGGCCTCCCGTT-3′ (710 bp 5′ fragment), and 5′-GCGAGAAGCCGTTCAAAT-3′ and 5′-TCTCTTTATACAATTGTGGTCCTAC-3′ (705 bp 3′ fragment) (two probes made separately and combined); *Tc-prd*, 5′-GAATACGGCCCTGTGTTATCT-3′ and 5′-ACCCATAGTACGGCTGATGT-3′ (1179 bp fragment); *Tc-wg*, 5′-CAACGCCAGAAGCAAGAAC-3′ and 5′-ACGACTTCCTGGGTACGATA-3′ (1095 bp fragment); *Tc-en*, 5′-TGCAAGTGGCTGAGTGT-3′ and 5′-GCAACTACGAGATTTGCCTTC-3′ (1001 bp fragment).

In the double *in situ* hybridisations in which *Tc-cad* mRNA is detected in red, the primary antibodies were switched such that anti-DIG-AP was used second (after anti-FITC-AP) to detect *Tc-cad DIG* probe, and signal was developed using INT/BCIP (see Schinko et al., 2009 for more details). Embryos were imaged on a Leica M165FC Fluorescence Stereo Microscope with a Q Imaging Retiga EXI colour cooled fluorescence camera and Q Capture Pro 7 software.

#### RNA interference (RNAi)

The *Tc-opa* gene is composed of two exons separated by a large 19.5 kb intron. A 710 bp DNA fragment corresponding to the first exon (i.e. template for 5′ dsRNA) was amplified by PCR from *Tribolium* cDNA using the following primer pair: 5′-CCCAAGAATGGCCTACTGC-3′ and 5′-TTGAAGGGCCTCCCGTT-3′. Similarly, a 705 bp DNA fragment corresponding to the 2nd exon (i.e. template for 3′ dsRNA) was amplified using the following primer pair: 5′-GCGAGAAGCCGTTCAAAT-3′ and 5′-TCTCTTTATACAATTGTGGTCCTAC-3′. These DNA fragments were cloned into the pGEM-Teasy vector (Promega) and antisense and sense ssRNA was produced using the T7 and SP6 MEGAscript High Yield Transcription Kits (Ambion). Antisense and sense ssRNA was then annealed in equimolar amounts and diluted to produce 1 μg/μl stocks of *Tc-opa* 5′ and 3′ dsRNA; these were aliquoted and stored at −20°C ready for future use.

Adult parental RNAi (pRNAi) was carried out using well-established protocols (Posnien et al., 2009). In the first round of pRNAi experiments, 250 females were injected with 5′ *Tc-opa* dsRNA, 245 females were injected with 3′ *Tc-opa* dsRNA and the two sets of parallel injection controls each involved injecting 240 females with control buffer. In the second round of pRNAi experiments, 100 females were used in each treatment (see Table S1 for more details). Following injection and a 2-day recovery, 48 or 72 h egg collections were obtained in white flour at regular intervals, with beetles ‘rested’ for 24 h on nutrient-rich wholemeal flour in between each egg collection. Roughly half of the eggs were immediately fixed for expression analysis, whereas the remainder were kept and allowed to develop for cuticle preparations. Embryonic RNAi (eRNAi) was performed by lightly bleaching 1- to 3-h-old eggs for 90 s in a 5% thin bleach solution. The eggs were then transferred to microscope slides (∼60 eggs per slide) and lined up along one edge of the slide ready for injection. Eggs were orientated so that they could be injected into the posterior pole, perpendicular to the egg axis, in rapid sequence. Eggs were injected with dsRNA or buffer using pulled needles made from borosilicate glass capillaries (Harvard Apparatus; GC100F-10; Part No. 30-0019). The needles were pulled using a Narishige needle puller (model PD-5), with the needle sharpened and standardized as much as possible using a Narishige needle grinder (model EG-45). Injections were carried out on a Zeiss Axiovert 10 inverted microscope, using a continuous flow injection set up.

## Supplementary Material

Supplementary information

## References

[DEV155580C1] Agathocleous M., Iordanova I., Willardsen M. I., Xue X. Y., Vetter M. L., Harris W. A. and Moore K. B. (2009). A directional Wnt/beta-catenin-Sox2-proneural pathway regulates the transition from proliferation to differentiation in the Xenopus retina. *Development*, 136, 3289-3299. 10.1242/dev.040451 19736324 PMC2739145

[DEV155580C2] Akam M. (1987). The molecular basis for metameric pattern in the Drosophila embryo. *Development* 101, 1-22. 2896587

[DEV155580C3] Akam M. (1994). Insect development. Is pairing the rule? *Nature* 367, 410-411. 8107797 10.1038/367410a0

[DEV155580C4] Aleksic J., Ferrero E., Fischer B., Shen S. P. and Russell S. (2013). The role of Dichaete in transcriptional regulation during Drosophila embryonic development. *BMC Genomics* 14, 861 10.1186/1471-2164-14-861 24314314 PMC3866562

[DEV155580C5] Ali R. G., Bellchambers H. M. and Arkell R. M. (2012). Zinc fingers of the cerebellum (Zic): transcription factors and co-factors. *International Journal of Biochemistry and Cell Biology* 44, 2065-2068. 10.1016/j.biocel.2012.08.012 22964024

[DEV155580C6] Andrioli L. P., Oberstein A. L., Corado M. S. G., Yu D. and Small S. (2004). Groucho-dependent repression by Sloppy-paired 1 differentially positions anterior pair-rule stripes in the Drosophila embryo. *Dev. Biol.* 276, 541-551. 10.1016/j.ydbio.2004.09.025 15581884

[DEV155580C7] Baumgartner S. and Noll M. (1990). Network of interactions among pair-rule genes regulating paired expression during primordial segmentation of Drosophila. *Mech. Dev.* 33, 1-18. 10.1016/0925-4773(90)90130-E 1982920

[DEV155580C8] Benedyk M. J., Mullen J. R. and DiNardo S. (1994). Odd-paired: a zinc finger pair-rule protein required for the timely activation of engrailed and wingless in Drosophila embryos. *Genes Dev.* 8, 105-117. 10.1101/gad.8.1.105 8288124

[DEV155580C9] Benton M. A., Pechmann M., Frey N., Stappert D., Conrads K. H., Chen Y.-T., Stamataki E., Pavlopoulos A. and Roth S. (2016). Toll genes have an ancestral role in axis elongation. *Curr. Biol.* 26, 1609-1615. 10.1016/j.cub.2016.04.055 27212406

[DEV155580C10] Bouchard M., St-Amand J. and Cote S. (2000). Combinatorial activity of pair-rule proteins on the Drosophila gooseberry early enhancer. *Dev. Biol.* 222, 135-146. 10.1006/dbio.2000.9702 10885752

[DEV155580C11] Brena C. and Akam M. (2013). An analysis of segmentation dynamics throughout embryogenesis in the centipede Strigamia maritima. *BMC Biol.* 11, 112 10.1186/1741-7007-11-112 24289308 PMC3879059

[DEV155580C12] Campos-Ortega J. A. and Hartenstein V. (1985). *The Embryonic Development of Drosophila Melanogaster*. Berlin/Heidelberg: Springer-Verlag.

[DEV155580C13] Cast A. E., Gao C., Amack J. D. and Ware S. M. (2012). An essential and highly conserved role for Zic3 in left-right patterning, gastrulation and convergent extension morphogenesis. *Dev. Biol.* 364, 22-31. 10.1016/j.ydbio.2012.01.011 22285814 PMC3294024

[DEV155580C14] Chan D. W., Liu V. W. S., Leung L. Y., Yao K. M., Chan K. K. L., Cheung A. N. Y. and Ngan H. Y. S., (2011). Zic2 synergistically enhances Hedgehog signalling through nuclear retention of Gli1 in cervical cancer cells. *J. Pathol.* 225, 525-534. 10.1002/path.2901 21661123

[DEV155580C15] Chen H., Xu Z., Mei C., Yu D. and Small S. (2012). A system of repressor gradients spatially organizes the boundaries of bicoid-dependent target genes. *Cell* 149, 618-629. 10.1016/j.cell.2012.03.018 22541432 PMC3535481

[DEV155580C16] Chervenak A. P., Hakim I. S. and Barald K. F. (2013). Spatiotemporal expression of zic genes during vertebrate inner ear development. *Dev. Dyn.* 242, 897-908. 10.1002/dvdy.23978 23606270 PMC3771324

[DEV155580C17] Chipman A. D. (2010). Parallel evolution of segmentation by co-option of ancestral gene regulatory networks. *BioEssays* 32, 60-70. 10.1002/bies.200900130 20020480

[DEV155580C18] Choe C. P. and Brown S. J. (2007). Evolutionary flexibility of pair-rule patterning revealed by functional analysis of secondary pair-rule genes, paired and sloppy-paired in the short-germ insect, Tribolium castaneum. *Dev. Biol.* 302, 281-294. 10.1016/j.ydbio.2006.09.037 17054935 PMC1800430

[DEV155580C19] Choe C. P., Miller S. C. and Brown S. J. (2006). A pair-rule gene circuit defines segments sequentially in the short-germ insect Tribolium castaneum. *Proc. Natl. Acad. Sci. U.S.A.* 103, 6560-6564. 10.1073/pnas.0510440103 16611732 PMC1564201

[DEV155580C20] Choe C. P., Stellabotte F. and Brown S. J., (2017). Regulation and function of odd-paired in Tribolium segmentation. *Dev. Genes Evol.* 10.1007/s00427-017-0590-7 28791475

[DEV155580C21] Clark E., (2017). Dynamic patterning by the Drosophila pair-rule network reconciles long-germ and short-germ segmentation C. Desplan, ed. *PLoS Biol.* 15, e2002439 10.1371/journal.pbio.2002439 28953896 PMC5633203

[DEV155580C22] Clark E. and Akam M. (2016a). Odd-paired controls frequency doubling in Drosophila segmentation by altering the pair-rule gene regulatory network. *eLife* 5, e18215 10.7554/eLife.18215 27525481 PMC5035143

[DEV155580C23] Clark E. and Akam M. (2016b). Data from: Odd-paired controls frequency doubling in Drosophila segmentation by altering the pair-rule gene regulatory network. *Dryad Digital Repository*. https://doi.org/10.5061/dryad.cg35k 10.5061/dryad.cg35k. PMC5035143 27525481

[DEV155580C24] Cooke J. and Zeeman E. C. (1976). A clock and wavefront model for control of the number of repeated structures during animal morphogenesis. *J. Theor. Biol.* 58, 455-476. 10.1016/S0022-5193(76)80131-2 940335

[DEV155580C25] Copf T., Schröder R. and Averof M. (2004). Ancestral role of caudal genes in axis elongation and segmentation. *Proc. Natl. Acad. Sci. U.S.A.* 101, 17711-17715. 10.1073/pnas.0407327102 15598743 PMC539741

[DEV155580C26] Damen W. G. M. (2002). Parasegmental organization of the spider embryo implies that the parasegment is an evolutionary conserved entity in arthropod embryogenesis. *Development* 129, 1239-1250. 11874919 10.1242/dev.129.5.1239

[DEV155580C27] Davis G. K. and Patel N. H. (2002). Short, long, and beyond: molecular and embryological approaches to insect segmentation. *Annu. Rev. Entomol.* 47, 669-699. 10.1146/annurev.ento.47.091201.145251 11729088

[DEV155580C28] DiNardo S. and O'Farrell P. H. (1987). Establishment and refinement of segmental pattern in the Drosophila embryo: spatial control of engrailed expression by pair-rule genes. *Genes Dev.* 1, 1212-1225. 10.1101/gad.1.10.1212 3123316

[DEV155580C29] DiNardo S., Heemskerk J., Dougan S. and O'Farrell P. H. (1994). The making of a maggot: patterning the Drosophila embryonic epidermis. *Curr. Opin Genet. Dev.* 4, 529-534. 10.1016/0959-437X(94)90068-E 7950320 PMC2873142

[DEV155580C30] Doe C. Q., Smouse D. and Goodman C. S. (1988). Control of neuronal fate by the Drosophila segmentation gene even-skipped. *Nature* 333, 376-378. 10.1038/333376a0 3374572

[DEV155580C31] Dönitz J., Schmitt-Engel C., Grossmann D., Gerischer L., Tech M., Schoppmeier M., Klingler M. and Bucher G. (2015). iBeetle-Base: a database for RNAi phenotypes in the red flour beetle Tribolium castaneum. *Nucleic Acids Res.* 43, D720-D725. 10.1093/nar/gku1054 25378303 PMC4383896

[DEV155580C32] El-Sherif E., Averof M. and Brown S. J. (2012). A segmentation clock operating in blastoderm and germband stages of Tribolium development. *Development* 139, 4341-4346. 10.1242/dev.085126 23095886 PMC3509729

[DEV155580C33] El-Sherif E., Zhu X., Fu J. and Brown S. J., (2014). Caudal regulates the spatiotemporal dynamics of pair-rule waves in tribolium. *PLoS Genet.* 10, e1004677 10.1371/journal.pgen.1004677 25329152 PMC4199486

[DEV155580C34] Eriksson B. J., Ungerer P. and Stollewerk A. (2013). The function of Notch signalling in segment formation in the crustacean Daphnia magna (Branchiopoda). *Dev. Biol.* 383, 321-330. 10.1016/j.ydbio.2013.09.021 24063806

[DEV155580C35] Farzana L. and Brown S. J. (2008). Hedgehog signaling pathway function conserved in Tribolium segmentation. *Dev. Genes. Evol.* 218, 181-192. 10.1007/s00427-008-0207-2 18392879 PMC2292471

[DEV155580C36] Fujimi T. J., Hatayama M. and Aruga J. (2012). Xenopus Zic3 controls notochord and organizer development through suppression of the Wnt/β-catenin signaling pathway. *Dev. Biol.* 361, 220-231. 10.1016/j.ydbio.2011.10.026 22056782

[DEV155580C37] Fujioka M., Emi-Sarker Y., Yusibova G. L., Goto T. and Jaynes J. B. (1999). Analysis of an even-skipped rescue transgene reveals both composite and discrete neuronal and early blastoderm enhancers, and multi-stripe positioning by gap gene repressor gradients. *Development* 126, 2527-2538. 10226011 10.1242/dev.126.11.2527 PMC2778309

[DEV155580C38] Graham A., Butts T., Lumsden A. and Kiecker C., (2014). What can vertebrates tell us about segmentation? *EvoDevo* 5, 24 10.1186/2041-9139-5-24 25009737 PMC4088296

[DEV155580C39] Green J. and Akam M. (2013). Evolution of the pair rule gene network: insights from a centipede. *Dev. Biol.* 382, 235-245. 10.1016/j.ydbio.2013.06.017 23810931 PMC3807789

[DEV155580C40] Gutjahr T., Vanario-Alonso C. E., Pick L. and Noll M. (1994). Multiple regulatory elements direct the complex expression pattern of the Drosophila segmentation gene paired. *Mech. Dev.* 48, 119-128. 10.1016/0925-4773(94)90021-3 7873402

[DEV155580C41] Häder T., La Rosée A., Ziebold U., Busch M., Taubert H., Jäckle H. and Rivera-Pomar R. (1998). Activation of posterior pair-rule stripe expression in response to maternal caudal and zygotic knirps activities. *Mech. Dev.* 71, 177-186. 10.1016/S0925-4773(98)00014-8 9507113

[DEV155580C42] Hafen E., Kuroiwa A. and Gehring W. J. (1984). Spatial distribution of transcripts from the segmentation gene fushi tarazu during Drosophila embryonic development. *Cell* 37, 833-841. 10.1016/0092-8674(84)90418-5 6430568

[DEV155580C43] Houtmeyers R., Souopgui J., Tejpar S. and Arkell R. (2013). The ZIC gene family encodes multi-functional proteins essential for patterning and morphogenesis. *Cell. Mol. Life Sci.* 70, 3791-3811. 10.1007/s00018-013-1285-5 23443491 PMC11113920

[DEV155580C44] Hunnekuhl V. S. and Akam M. (2017). Formation and subdivision of the head field in the centipede Strigamia maritima, as revealed by the expression of head gap gene orthologues and hedgehog dynamics. *EvoDevo* 8, 18 10.1186/s13227-017-0082-x 29075435 PMC5654096

[DEV155580C45] Inoue T., Ota M., Mikoshiba K. and Aruga J. (2007). Zic2 and Zic3 synergistically control neurulation and segmentation of paraxial mesoderm in mouse embryo. *Dev. Biol.* 306, 669-684. 10.1016/j.ydbio.2007.04.003 17490632

[DEV155580C46] Isshiki T., Pearson B., Holbrook S. and Doe C. Q. (2001). Drosophila neuroblasts sequentially express transcription factors which specify the temporal identity of their neuronal progeny. *Cell* 106, 511-521. 10.1016/S0092-8674(01)00465-2 11525736

[DEV155580C47] Jaeger J. (2011). The gap gene network. *Cell. Mol. Life Sci.* 68, 243-274. 10.1007/s00018-010-0536-y 20927566 PMC3016493

[DEV155580C48] Janssen R. and Budd G. E. (2013). Deciphering the onychophoran “segmentation gene cascade”: gene expression reveals limited involvement of pair rule gene orthologs in segmentation, but a highly conserved segment polarity gene network. *Dev. Biol.* 382, 224-234. 10.1016/j.ydbio.2013.07.010 23880430

[DEV155580C49] Janssen R., Budd G. E., Prpic N.-M. and Damen W. G. M. (2011). Expression of myriapod pair rule gene orthologs. *EvoDevo* 2, 5 10.1186/2041-9139-2-5 21352542 PMC3058060

[DEV155580C50] Jürgens G., Wieschaus E., Nüsslein-Volhard C. and Kluding H. (1984). Mutations affecting the pattern of the larval cuticle in Drosophila melanogaster II. Zygotic loci on the third chromosome. *Wilehm Roux Arch Dev Biol.* 193, 283-295. 10.1007/BF00848157 28305338

[DEV155580C51] Kanayama M., Akiyama-Oda Y., Nishimura O., Tarui H., Agata K. and Oda H. (2011). Travelling and splitting of a wave of hedgehog expression involved in spider-head segmentation. *Nat. Commun.* 2, 500 10.1038/ncomms1510 21988916 PMC3207210

[DEV155580C52] Kilchherr F., Baumgartner S., Bopp D., Frei E. and Noll M. (1986). Isolation of the paired gene of Drosophila and its spatial expression during early embryogenesis. *Nature* 321, 493-499. 10.1038/321493a0

[DEV155580C53] Kormish J. D., Sinner D. and Zorn A. M. (2010). Interactions between SOX factors and Wnt/beta-catenin signaling in development and disease. *Dev. Dyn.* 239, 56-68. 10.1002/dvdy.22046 19655378 PMC3269784

[DEV155580C54] Kosman D., Mizutani C., Lemons D., Cox W., McGinnis W. and Bier E. (2004). Multiplex detection of RNA expression in drosophila embryos. *Science* 305, 846-846. 10.1126/science.1099247 15297669

[DEV155580C55] Koyabu Y., Nakata K., Mizugishi K., Aruga J. and Mikoshiba K. (2001). Physical and functional interactions between zic and gli proteins. *J. Biol. Chem.* 276, 6889-6892. 10.1074/jbc.C000773200 11238441

[DEV155580C56] Krause G. (1939). Die Eytipen der Insekten. *Biol. Zbl.* 59, 495-536.

[DEV155580C57] Kuhn D. T., Turenchalk G., Mack J. A., Packert G. and Kornberg T. B. (1995). Analysis of the genes involved in organizing the tail segments of the Drosophila melanogaster embryo. *Mech. Dev.* 53, 3-13. 10.1016/0925-4773(95)00399-1 8555109

[DEV155580C58] Kuhn D. T., Chaverri J. M., Persaud D. A. and Madjidi A. (2000). Pair-rule genes cooperate to activate en stripe 15 and refine its margins during germ band elongation in the D. melanogaster embryo. *Mech. Dev.* 95, 297-300. 10.1016/S0925-4773(00)00358-0 10906481

[DEV155580C59] La Rosée A., Häder T., Taubert H., Rivera-Pomar R. and Jäckle H., (1997). Mechanism and Bicoid-dependent control of hairy stripe 7 expression in the posterior region of the Drosophila embryo. *EMBO J.* 16, 4403-4411. 10.1093/emboj/16.14.4403 9250684 PMC1170066

[DEV155580C60] Liu P. Z. and Kaufman T. C. (2005). Short and long germ segmentation: unanswered questions in the evolution of a developmental mode. *Evol. Dev.* 7, 629-646. 10.1111/j.1525-142X.2005.05066.x 16336416

[DEV155580C61] Lynch J. A., El-Sherif E. and Brown S. J. (2012). Comparisons of the embryonic development of Drosophila, Nasonia, and Tribolium. *Wiley Interdiscip. Rev. Dev. Biol.* 1, 16-39. 10.1002/wdev.3 23801665 PMC5323069

[DEV155580C62] Ma Y., Niemitz E. L., Nambu P. A., Shan X., Sackerson C., Fujioka M., Goto T. and Nambu J. R. (1998). Gene regulatory functions of Drosophila Fish-hook, a high mobility group domain Sox protein. *Mech. Dev.* 73, 169-182. 10.1016/S0925-4773(98)00050-1 9622621

[DEV155580C63] MacArthur S., Li X.-Y., Li J., Brown J. B., Chu H. C., Zeng L., Grondona B. P., Hechmer A., Simirenko L., Keränen S. V. E. et al. (2009). Developmental roles of 21 Drosophila transcription factors are determined by quantitative differences in binding to an overlapping set of thousands of genomic regions. *Genome Biol.* 10, R80 10.1186/gb-2009-10-7-r80 19627575 PMC2728534

[DEV155580C64] Macdonald P. M. and Struhl G. (1986). A molecular gradient in early Drosophila embryos and its role in specifying the body pattern. *Nature* 324, 537-545. 10.1038/324537a0 2878369

[DEV155580C65] Manoukian A. S. and Krause H. M. (1992). Concentration-dependent activities of the even-skipped protein in Drosophila embryos. *Genes Dev.* 6, 1740-1751. 10.1101/gad.6.9.1740 1355458

[DEV155580C66] McGregor A. P., Pechmann M., Schwager E. E. and Damen W. G. M. (2009). An ancestral regulatory network for posterior development in arthropods. *Communicative Integrative Biol.* 2, 174-176. 10.4161/cib.7710 PMC2686376 19513274

[DEV155580C67] Mito T., Kobayashi C., Sarashina I., Zhang H., Shinahara W., Miyawaki K., Shinmyo Y., Ohuchi H. and Noji S. (2007). even-skipped has gap-like, pair-rule-like, and segmental functions in the cricket Gryllus bimaculatus, a basal, intermediate germ insect (Orthoptera). *Dev. Biol.* 303, 202-213. 10.1016/j.ydbio.2006.11.003 17174947

[DEV155580C68] Mlodzik M., Gibson G. and Gehring W. J. (1990). Effects of ectopic expression of caudal during drosophila development. *Development* 109, 271-277. 1976085 10.1242/dev.109.2.271

[DEV155580C69] Mondal S., Ivanchuk S. M., Rutka J. T. and Boulianne G. L. (2007). Sloppy paired 1/2 regulate glial cell fates by inhibiting Gcm function. *Glia* 55, 282-293. 10.1002/glia.20456 17091489

[DEV155580C70] Murgan S., Kari W., Rothbächer U., Iché-Torres M., Mélénec P., Hobert O. and Bertrand V. (2015). Atypical Transcriptional activation by TCF via a Zic transcription factor in C. elegans neuronal precursors. *Dev. Cell* 33, 737-745. 10.1016/j.devcel.2015.04.018 26073017 PMC4480195

[DEV155580C71] Nakamoto A., Hester S. D., Constantinou S. J., Blaine W. G., Tewksbury A. B., Matei M. T., Nagy L. M. and Williams T. A. (2015). Changing cell behaviours during beetle embryogenesis correlates with slowing of segmentation. *Nat. Commun.* 6, 6635 10.1038/ncomms7635 25858515

[DEV155580C72] Nakao H., (2017). A Bombyx homolog of ovo is a segmentation gene that acts downstream of Bm-wnt1(Bombyx wnt1 homolog). *Gene Expr. Patterns* 27, 1-7. 10.1016/j.gep.2017.10.002 28988845

[DEV155580C73] Nambu P. A. and Nambu J. R. (1996). The Drosophila fish-hook gene encodes a HMG domain protein essential for segmentation and CNS development. *Development* 122, 3467-3475. 8951062 10.1242/dev.122.11.3467

[DEV155580C74] Nasiadka A., Dietrich B. H. and Krause H. M. (2002). Anterior – posterior patterning in the Drosophila embryo. *Adv. Dev. Biol. Biochem.* 12, 155-204. 10.1016/S1569-1799(02)12027-2

[DEV155580C75] Neijts R., Amin S., van Rooijen C., and Deschamps J. (2016). Cdx is crucial for the timing mechanism driving colinear Hox activation and defines a trunk segment in the Hox cluster topology. *Dev. Biol.* 422, 146-154. 10.1016/j.ydbio.2016.12.024 28041967

[DEV155580C76] Nüsslein-Volhard C. and Wieschaus E. (1980). Mutations affecting segment number and polarity in Drosophila. *Nature* 287, 795-801. 10.1038/287795a0 6776413

[DEV155580C77] Oates A. C., Morelli L. G. and Ares S., (2012). Patterning embryos with oscillations: structure, function and dynamics of the vertebrate segmentation clock. *Development* 139, 625-639. 10.1242/dev.063735 22274695

[DEV155580C78] Oberhofer G., Grossmann D., Siemanowski J. L., Beissbarth T. and Bucher G. (2014). Wnt/ -catenin signaling integrates patterning and metabolism of the insect growth zone. *Development* 141, 4740-4750. 10.1242/dev.112797 25395458 PMC4299277

[DEV155580C79] Ochoa-Espinosa A., Yucel G., Kaplan L., Pare A., Pura N., Oberstein A., Papatsenko D. and Small S. (2005). The role of binding site cluster strength in Bicoid-dependent patterning in Drosophila. *Proc. Natl. Acad. Sci. USA* 102, 4960-4965. 10.1073/pnas.0500373102 15793007 PMC555997

[DEV155580C80] Olesnicky E. C., Brent A. E., Tonnes L., Walker M., Pultz M. A., Leaf D. and Desplan C. (2006). A caudal mRNA gradient controls posterior development in the wasp Nasonia. *Development* 133, 3973-3982. 10.1242/dev.02576 16971471

[DEV155580C81] Palmeirim I., Henrique D., Ish-Horowicz D. and Pourquié O. (1997). Avian hairy gene expression identifies a molecular clock linked to vertebrate segmentation and somitogenesis. *Cell* 91, 639-648. 10.1016/S0092-8674(00)80451-1 9393857

[DEV155580C82] Patel N. H. (1994). The evolution of arthropod segmentation: insights from comparisons of gene expression patterns. *Dev. Suppl.* 201-207. 7579520

[DEV155580C83] Patel N. H., Condron B. G. and Zinn K. (1994). Pair-rule expression patterns of even-skipped are found in both short- and long-germ beetles. *Nature* 367, 429-434. 10.1038/367429a0 8107801

[DEV155580C84] Peel A. and Akam M. (2003). Evolution of segmentation: rolling back the clock. *Curr. Biol.* 13, 708-710. 10.1016/j.cub.2003.08.045 13678609

[DEV155580C85] Peel A. D. and Averof M. (2010). Early asymmetries in maternal transcript distribution associated with a cortical microtubule network and a polar body in the beetle Tribolium castaneum. *Dev. Dyn.* 239, 2875-2887. 10.1002/dvdy.22423 20857499

[DEV155580C86] Peel A. D., Chipman A. D. and Akam M. (2005). Arthropod segmentation: beyond the Drosophila paradigm. *Nat. Rev. Genet.* 6, 905-916. 10.1038/nrg1724 16341071

[DEV155580C87] Posnien N., Schinko J., Grossmann D., Shippy T. D., Konopova B. and Bucher G. (2009). RNAi in the red flour beetle (Tribolium). *Cold Spring Harb. Protoc.* 4, 1-9. 10.1101/pdb.prot5256 20147232

[DEV155580C88] Pourebrahim R., Houtmeyers R., Ghogomu S., Janssens S., Thelie A., Tran H. T., Langenberg T., Vleminckx K., Bellefroid E. and Cassiman J. J. et al., (2011). Transcription factor Zic2 inhibits Wnt/β-catenin protein signaling. *J. Biol. Chem.* 286, 37732-37740. 10.1074/jbc.M111.242826 21908606 PMC3199516

[DEV155580C89] Pueyo J. I., Lanfear R. and Couso J. P. (2008). Ancestral Notch-mediated segmentation revealed in the cockroach Periplaneta americana. *Proc. Natl. Acad. Sci. U.S.A.* 105, 16614-16619. 10.1073/pnas.0804093105 18927236 PMC2568983

[DEV155580C90] Quinn M. E., Haaning A. and Ware S. M. (2012). Preaxial polydactyly caused by Gli3 haploinsufficiency is rescued by Zic3 loss of function in mice. *Hum. Mol. Genet.* 21, 1888-1896. 10.1093/hmg/dds002 22234993 PMC3313802

[DEV155580C91] Rivera-Pomar R., Lu X., Perrimon N., Taubert H. and Jäckle H. (1995). Activation of posterior gap gene expression in the Drosophila blastoderm. *Nature* 376, 253-256. 10.1038/376253a0 7617036

[DEV155580C92] Rosenberg M. I., Brent A. E., Payre F. and Desplan C. (2014). Dual mode of embryonic development is highlighted by expression and function of Nasonia pair-rule genes. *Elife* 3, e01440 10.7554/eLife.01440 24599282 PMC3941026

[DEV155580C93] Russell S. R., Sanchez-Soriano N., Wright C. R. and Ashburner M. (1996). The Dichaete gene of Drosophila melanogaster encodes a SOX-domain protein required for embryonic segmentation. *Development* 122, 3669-3676. 8951082 10.1242/dev.122.11.3669

[DEV155580C94] Sander K. (1976). Specification of the basic body pattern in insect embryogenesis. *Adv. Insect Physiol.* 12, 125-238. 10.1016/S0065-2806(08)60255-6

[DEV155580C95] Sanek N. A., Taylor A. A., Nyholm M. K. and Grinblat Y. (2009). Zebrafish zic2a patterns the forebrain through modulation of Hedgehog-activated gene expression. *Development* 136, 3791-3800. 10.1242/dev.037820 19855021 PMC2766342

[DEV155580C96] Sarrazin A. F., Peel A. D. and Averof M. (2012). A segmentation clock with two-segment periodicity in insects. *Science* 336, 338-341. 10.1126/science.1218256 22403177

[DEV155580C97] Schindelin J., Arganda-Carreras I., Frise E., Kaynig V., Longair M., Pietzsch T., Preibisch S., Rueden C., Saalfeld S., Schmid B. et al. (2012). Fiji: an open-source platform for biological-image analysis. *Nat. Methods* 9, 676-682. 10.1038/nmeth.2019 22743772 PMC3855844

[DEV155580C98] Schinko J. B., Kreuzer N., Offen N., Posnien N., Wimmer E. A. and Bucher G. (2008). Divergent functions of orthodenticle, empty spiracles and buttonhead in early head patterning of the beetle Tribolium castaneum (Coleoptera). *Dev. Biol.* 317, 600-613. 10.1016/j.ydbio.2008.03.005 18407258

[DEV155580C99] Schinko J., Posnien N., Kittelmann S., Koniszewski N. and Bucher G. (2009). Single and double whole-mount in situ hybridization in red flour beetle (Tribolium) embryos. *Cold Spring Harb. Protoc.* 4, 1-7. 10.1101/pdb.prot5258 20147234

[DEV155580C100] Schneider C. A., Rasband W. S. and Eliceiri K. W. (2012). NIH image to ImageJ: 25 years of image analysis. *Nat. Methods* 9, 671-675. 10.1038/nmeth.2089 22930834 PMC5554542

[DEV155580C101] Schönauer A., Paese C. L. B., Hilbrant M., Leite D. J., Schwager E. E., Feitosa N. M., Eibner C., Damen W.G. and McGregor A. P, (2016). The Wnt and Delta-Notch signalling pathways interact to direct pair-rule gene expression via caudal during segment addition in the spider Parasteatoda tepidariorum. *Development* 2455-2463. 10.1242/dev.131656 27287802

[DEV155580C102] Schroeder M. D., Greer C. and Gaul U. (2011). How to make stripes: deciphering the transition from non-periodic to periodic patterns in Drosophila segmentation. *Development* 138, 3067-3078. 10.1242/dev.062141 21693522 PMC3119311

[DEV155580C103] Schulz C. and Tautz D. (1995). Zygotic caudal regulation by hunchback and its role in abdominal segment formation of the Drosophila embryo. *Development* 121, 1023-1028. 7743918 10.1242/dev.121.4.1023

[DEV155580C104] Schulz C., Schröder R., Hausdorf B., Wolff C. and Tautz D. (1998). A caudal homologue in the short germ band beetle Tribolium shows similarities to both, the Drosophila and the vertebrate caudal expression patterns. *Dev. Genes Evol.* 208, 283-289. 10.1007/s004270050183 9683744

[DEV155580C105] Shimojo H., Ohtsuka T. and Kageyama R. (2008). Oscillations in notch signaling regulate maintenance of neural progenitors. *Neuron* 58, 52-64. 10.1016/j.neuron.2008.02.014 18400163

[DEV155580C106] Stapleton M., Carlson J., Brokstein P., Yu C., Champe M., George R., Guarin H., Kronmiller B., Pacleb J. and Park S., (2002). A Drosophila full-length cDNA resource. *Genome Biol.* 3, RESEARCH0080 10.1186/gb-2002-3-12-research0080 12537569 PMC151182

[DEV155580C107] Stollewerk A., Schoppmeier M. and Damen W. G. M. (2003). Involvement of Notch and Delta genes in spider segmentation. *Nature* 423, 863-865. 10.1038/nature01682 12815430

[DEV155580C108] Sucena É., Vanderberghe K., Zhurov V. and Grbi M., (2014). Reversion of developmental mode in insects : evolution from long germband to short germband in the polyembrionic wasp Macrocentrus cingulum Brischke. *Evol. Dev.* 246, 233-246. 10.1111/ede.12086 24981069

[DEV155580C109] Surkova S., Kosman D., Kozlov K., Manu, Myasnikova E., Samsonova A. A., Spirov A., Vanario-Alonso C. E., Samsonova M. and Reinitz J. (2008). Characterization of the Drosophila segment determination morphome. *Dev. Biol.* 313, 844-862. 10.1016/j.ydbio.2007.10.037 18067886 PMC2254320

[DEV155580C110] van Rooijen C., Simmini S., Bialecka M., Neijts R., van de Ven C., Beck F. and Deschamps J. (2012). Evolutionarily conserved requirement of Cdx for post-occipital tissue emergence. *Development* 139, 2576-2583. 10.1242/dev.079848 22675207

[DEV155580C111] Williams T. A. and Nagy L. M. (2016). Linking gene regulation to cell behaviors in the posterior growth zone of sequentially segmenting arthropods. *Arthropod. Struct. Dev.* 46, 380-394. 10.1016/j.asd.2016.10.003 27720841

[DEV155580C112] Wolfe J. M., Daley A. C., Legg D. A. and Edgecombe G. D. (2016). Fossil calibrations for the arthropod Tree of Life. *Earth-Sci. Rev.* 160, 43-110. 10.1016/j.earscirev.2016.06.008

[DEV155580C113] Wood H. B. and Episkopou V. (1999). Comparative expression of the mouse Sox1, Sox2 and Sox3 genes from pre-gastrulation to early somite stages. *Mech. Dev.* 86, 197-201. 10446282 10.1016/s0925-4773(99)00116-1

[DEV155580C114] Wymeersch F. J., Huang Y., Blin G., Cambray N., Wilkie R., Wong F. C.and Wilson V. (2016). Position-dependent plasticity of distinct progenitor types in the primitive streak. *Elife* 5, 1-28. 10.7554/eLife.10042 PMC4798969 26780186

[DEV155580C115] Zhao G. and Skeath J. B. (2002). The Sox-domain containing gene Dichaete/fish-hook acts in concert with vnd and ind to regulate cell fate in the Drosophila neuroectoderm. *Development* 129, 1165-1174. 11874912 10.1242/dev.129.5.1165

[DEV155580C116] Zhao G., Boekhoff-Falk G., Wilson B. A. and Skeath J. B., (2007). Linking pattern formation to cell-type specification: Dichaete and Ind directly repress achaete gene expression in the Drosophila CNS. *Proc. Natl. Acad. Sci. USA* 104, 3847-3852. 10.1073/pnas.0611700104 17360441 PMC1820672

[DEV155580C117] Zhu X., Rudolf H., Haeley L., François P., Brown S. J., Klingler M. and El-Sherif E. (2017). Speed regulation of genetic cascades allows for evolvability in the body plan specification of insects. *Proc. Natl. Acad. Sci. USA* 114, E8646-E8655. 10.1073/pnas.1702478114 28973882 PMC5642680

