# Supplementary material for: Evidence for the temporal regulation of insect segmentation by a conserved sequence of transcription factors
Source: Development. 2018 May 23;145(10):dev155580. doi: 10.1242/dev.155580 (PMC6001374; doi:10.1242/dev.155580)
Supplement: Supplementary information [file develop-145-155580-s1.pdf]

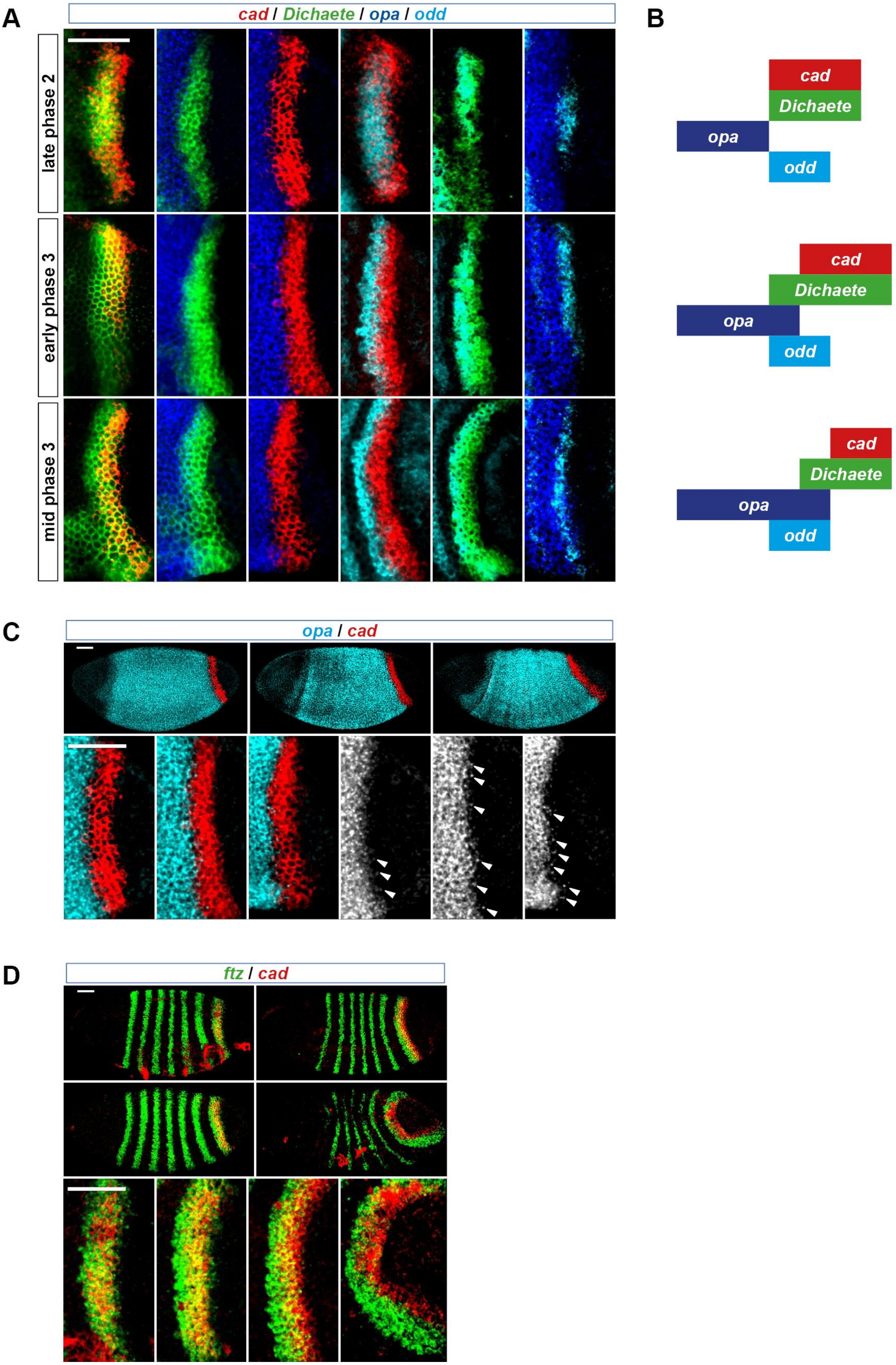

Development • Supplementary information

Fig. S1. Shifting boundaries of timing factor expression within the *Drosophila* tail.
(**A**) Cropped and rotated enlargements of the *odd* stripe 7 region, at three timepoints (equivalent to the 3 penultimate rows in 2A,B.) (**B**) Schematic diagram of the shifting expression domains within the tail, based on the images in (A). Expression boundaries of *cad*, *Dichaete*, and *opa* shift posteriorly across *odd* stripe 7 over time. The anterior boundary of *odd* stripe 7 is assumed to be static (Surkova et al. 2008; Clark & Akam 2016). (**C**) Nascent transcription (nuclear dots, marked by arrowheads) of *opa* expression are observed within the *cad* domain throughout gastrulation and early germband extension, indicating that the *opa* expression domain is expanding posteriorly. (**D**) The *cad* posterior domain shifts markedly relative to stripe 7 of *ftz* over time. Cropped and rotated enlargements of the stripe 7 region are show below the whole embryo views. The bright red regions of staining in (D) outside of the *cad* posterior domain are artefacts caused by bits of debris stuck to the embryos. Scale bars = 50 μm.

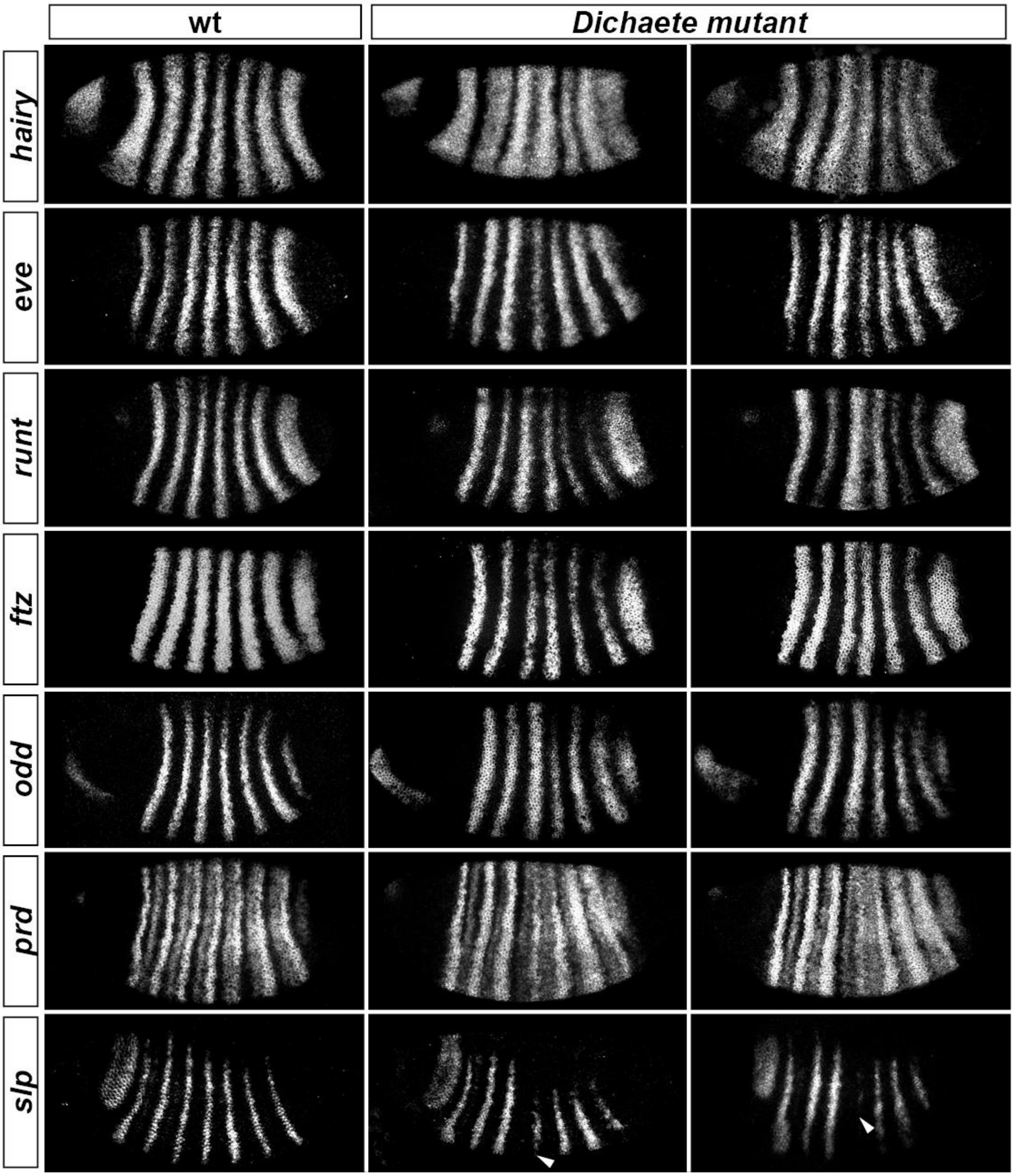

Fig. S2. Pair-rule gene expression in *Dichaete* mutant embryos.
Embryos are at late phase 2. In all cases, pair-rule periodicity is still present in the mutants, but stripes are irregular in width and intensity. Expression patterns are broadly consistent across stage-matched embryos. Arrowheads point to a weak/delayed *slp* stripe 4.

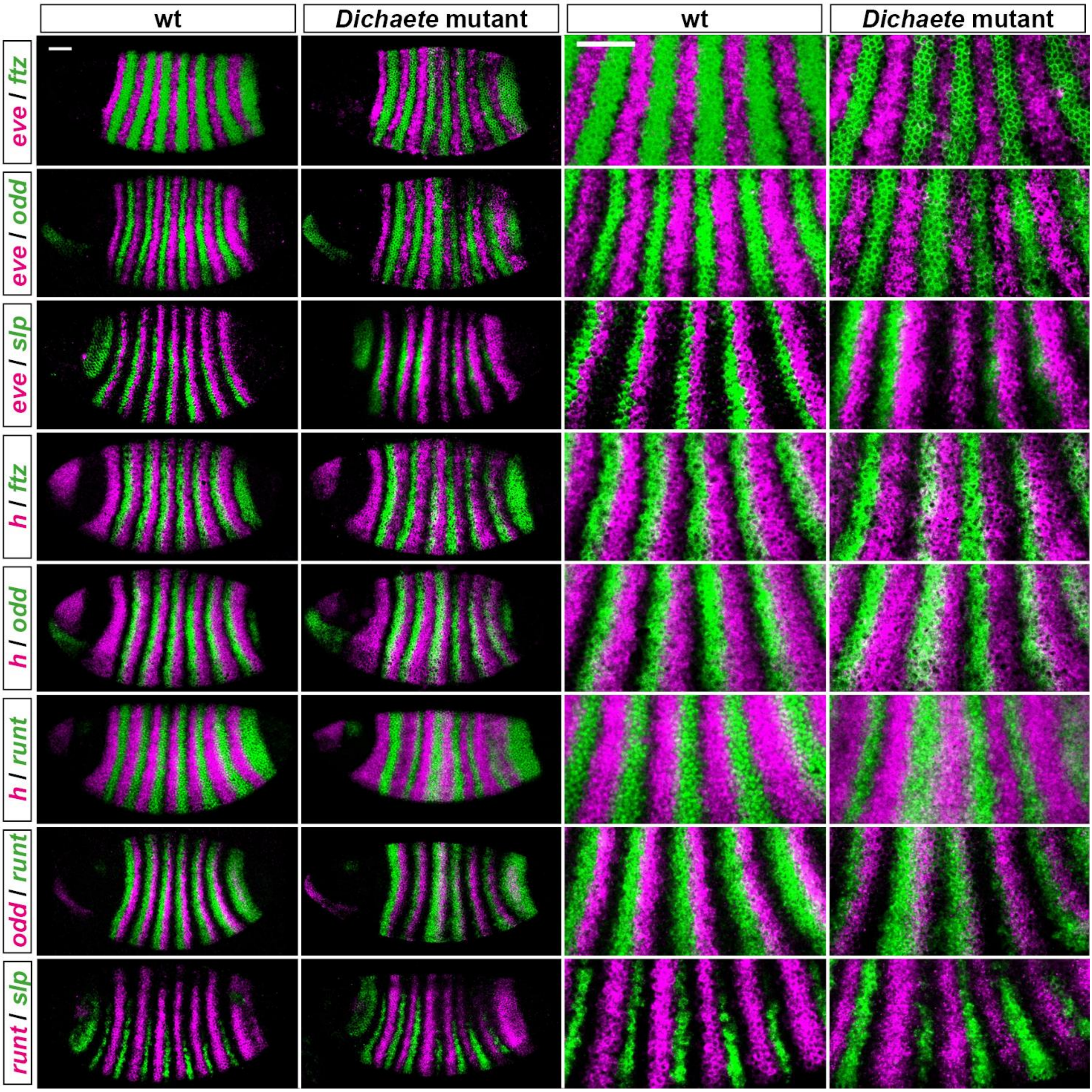

Fig. S3. Relative phasing of pair-rule stripes in *Dichaete* mutant embryos.
Embryos are at late phase 2. Expression patterns of repressors (magenta) are shown relative to those of their target genes (green). (For a description of the pair-rule network, see Clark (2017).) In most cases (e.g., *eve* versus *ftz*/*odd*/*slp*, or *runt* versus *slp*), the relative phasing of the stripes is preserved, suggesting that cross-regulatory interactions are operating normally. Only the phasing of *runt* expression relative to *hairy* and *odd* is clearly abnormal. Scale bars = 50 µm.

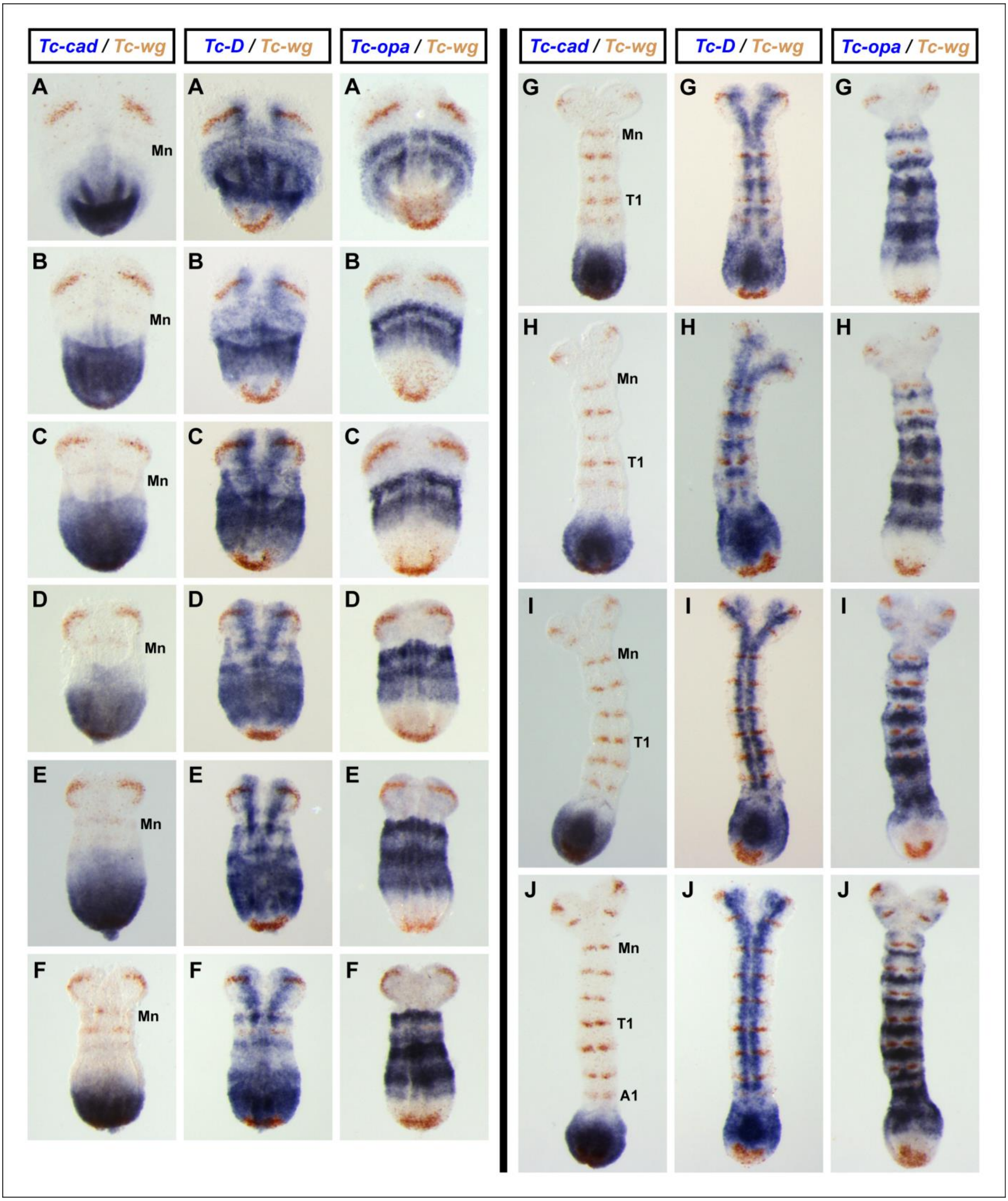

Development • Supplementary information

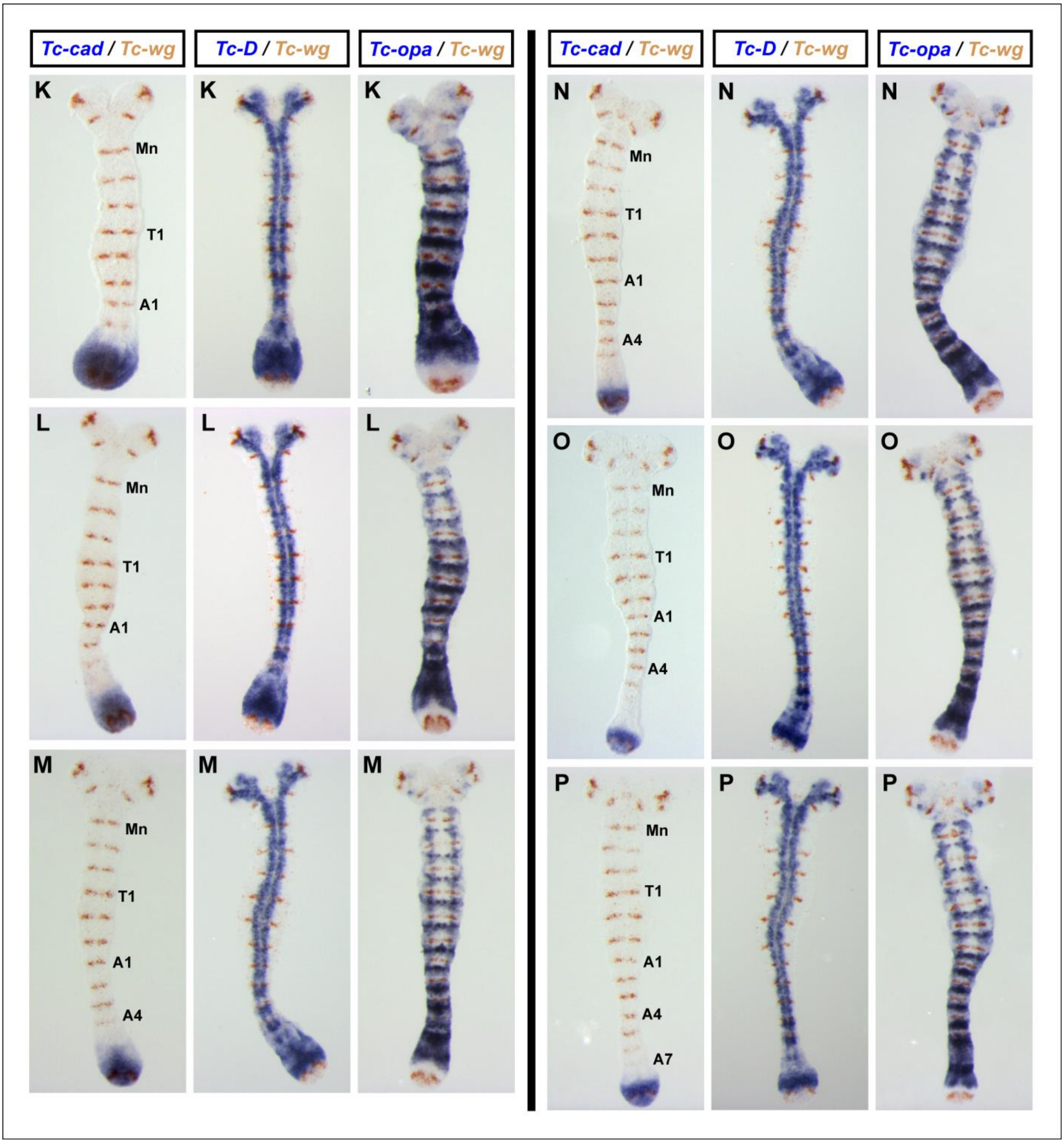

Development • Supplementary information

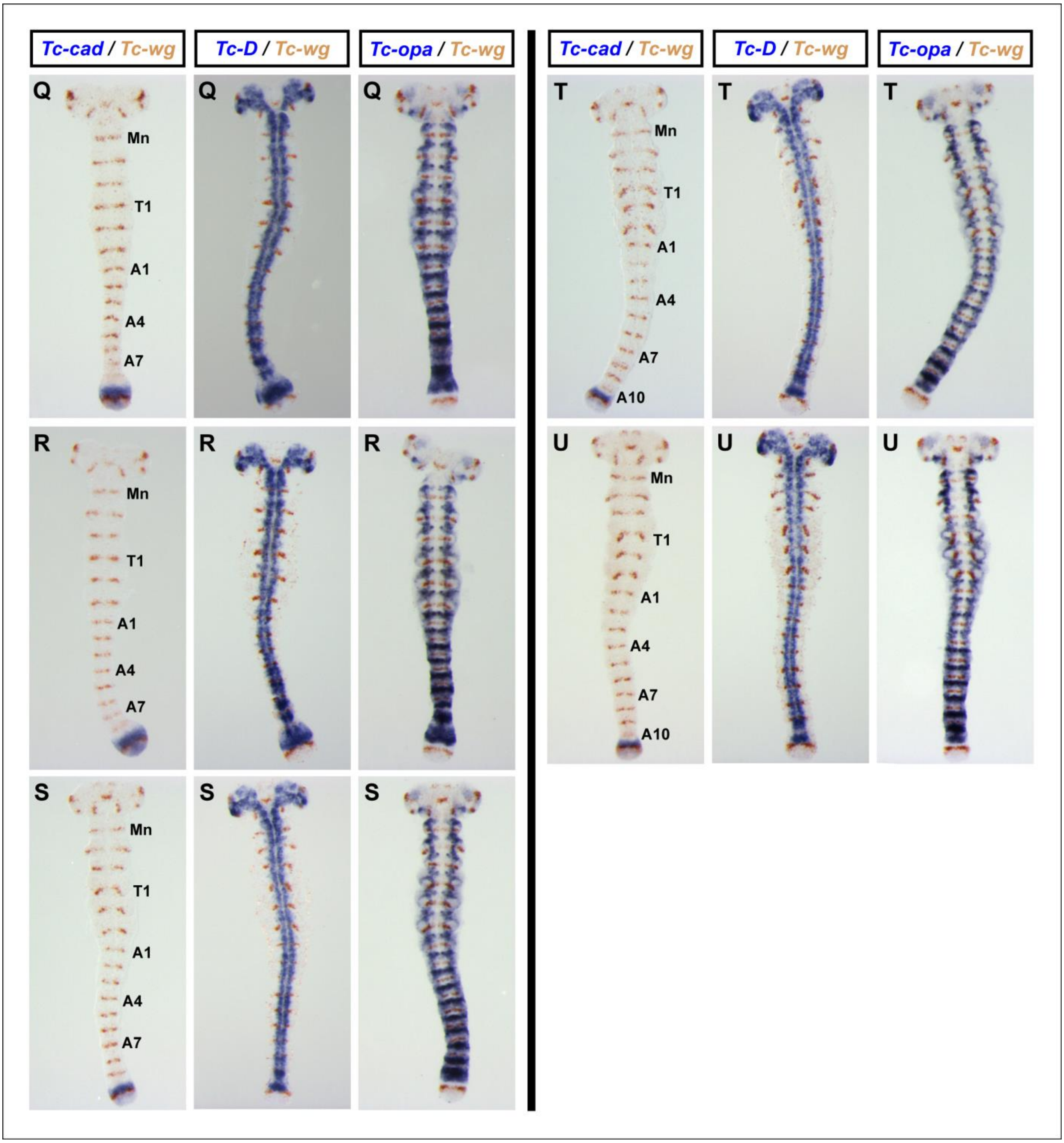

Fig. S4. Expression of *Tc-cad*, *Tc-Dichaete*, and *Tc-opa* relative to a common segment marker, *Tc-wg*, in *Tribolium castaneum* germband stage embryos.

(A-U). Sets of three *Tribolium castaneum* germband stage embryos that have been stage matched using *Tc-wg* expression patterns (*Tc-wg* expression brown in all panels). Stage-matched germband embryos increase in age from **A** to **U**. In each set of embryos, the left-hand embryo is also stained for *Tc-cad* expression, the middle embryo is stained for *Tc-Dichaete* expression and the right-hand embryo is stained for *Tc-opa* expression (all blue stains). In the double in situ hybridizations for *Tc-cad* & *Tc-wg* (left-hand embryos) the mandibular (Mn), prothoracic (T1), 1st abdominal (A1), 4th abdominal (A4), 7th abdominal (A7) and/or 10th abdominal (A10) stripes of *Tc-wg* expression have been labeled. Note how the relative position of the expression domains of these three genes is remarkable conserved across progressive germband elongation stages. Consult Fig. 5 for a clear comparison across different developmental stages, rather than between *Tc-cad*, *Tc-Dichaete* & *Tc-opa* expression patterns.

Development • Supplementary information

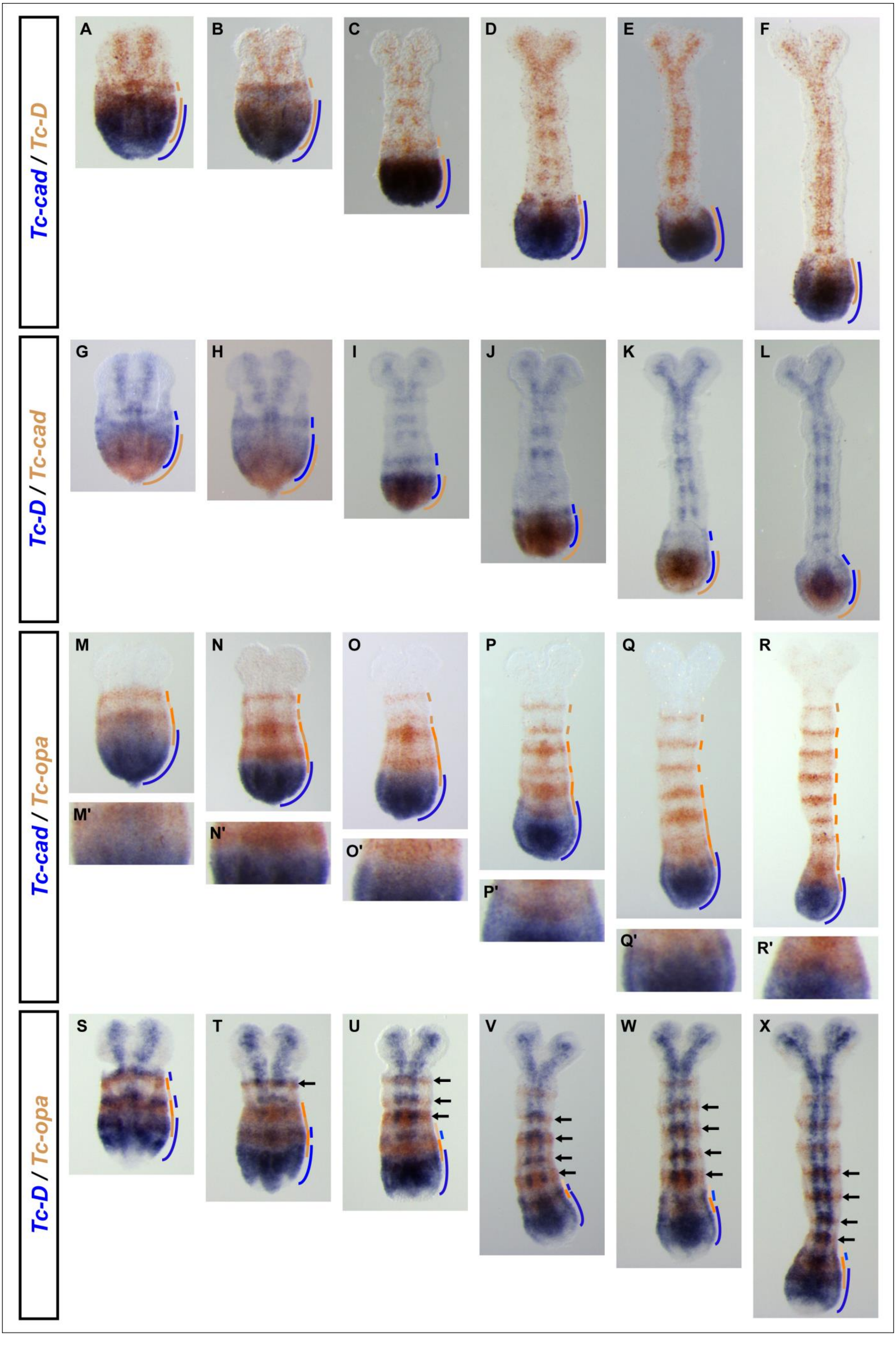

Development • Supplementary information

Fig S5. Expression patterns of *Tc-cad*, Tc-*Dichaete*, and *Tc-opa* in relation to each other in *Tribolium castaneum* germband stage embryos.

(**A-F**). Double in situ hybridization for *Tc-cad* (blue) and *Tc-Dichaete* (brown) in embryos of increasing age from left (A) to right (F). (**G-L**). As for panels (A-F), but this time *Tc-Dichaete* DIG and *Tc-cad* FITC RNA probes were used instead of *Tc-cad* DIG and *Tc-Dichaete* FITC RNA probes such that the colours are reversed. Note the stripe of *Tc-Dichaete* expression that is observed anterior to the *Tc-cad* domain in some, but not all, embryos. (**M-R**). Double in situ hybridization for *Tc-cad* (blue) and *Tc-opa* (brown) in embryos of increasing age from left (M) to right (N). Panels (M′-R′) show higher magnification images of the regions in M-R where *Tc-cad* and *Tc-opa* expression overlaps. These data suggest that as posterior germband cells move anteriorly relative to the posterior tip of the elongating embryo due to convergent extension cell movements, they experience a drop in *Tc-cad* expression levels as *Tc-opa* expression levels increase. (**S-X**). Double in situ hybridization for *Tc-Dichaete* (blue) and *Tc-opa* (brown) in embryos of increasing age from left (S) to right (X). Black arrows points to late *Tc-opa* segmental stripes that overlap strong segmentally-reiterated *Tc-Dichaete* expression domains that are limited to the medially positioned neuroectoderm. Colour-coded lines on the right-hand side of the embryos indicate our interpretations of the expression patterns in (A-X).

Development • Supplementary information

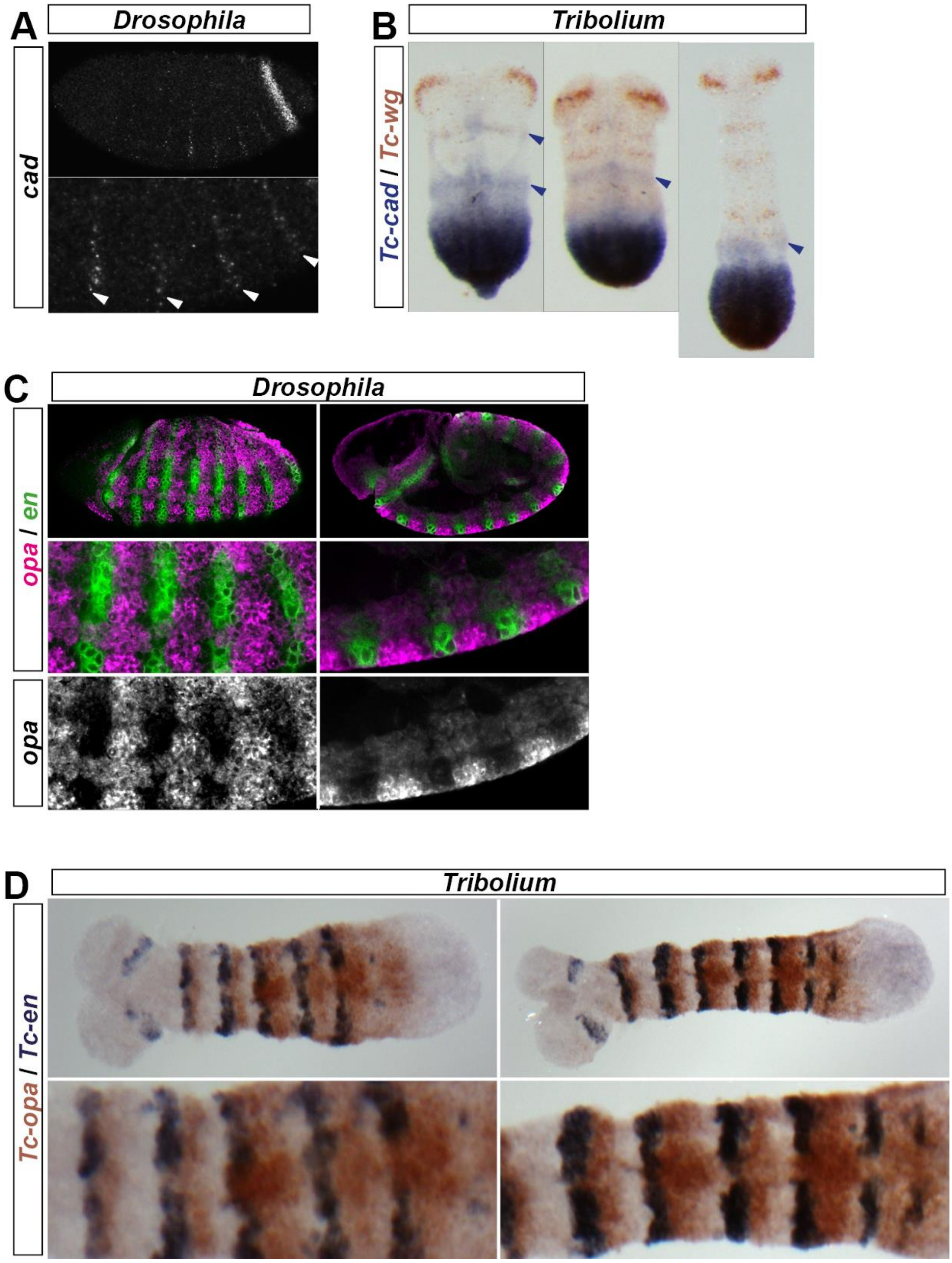

Fig. S6. Details of *cad* and *opa* expression in *Drosophila* are paralleled in *Tribolium*.
(**A**) At gastrulation, *cad* is transiently expressed in weak pair-rule stripes (white arrowheads). These stripes have previously been observed at the protein level during germband extension (Macdonald & Struhl 1986). (**B**) Weak pair-rule stripes of *Tc-cad* (blue arrowheads) are sometimes observed anterior to the broad posterior domain. The domain corresponding to the lower arrowhead in the left panel has been reported previously (Schulz & Tautz 1995). In both *Drosophila* and *Tribolium*, these pair-rule *cad* stripes are located in the posterior of even-numbered parasegments, overlapping with even-numbered *wg* stripes. (**C**) During germband extension, ventral *opa* expression transitions to a segmental pattern. *opa* stripes posteriorly abut each *en* stripe, but are excluded from the cell row anterior to each *en* stripe. (**D**) *Tc-opa* exhibits an equivalent pattern in the segmented germband, posteriorly abutting each *Tc-en* stripe (images from Fig. S10).

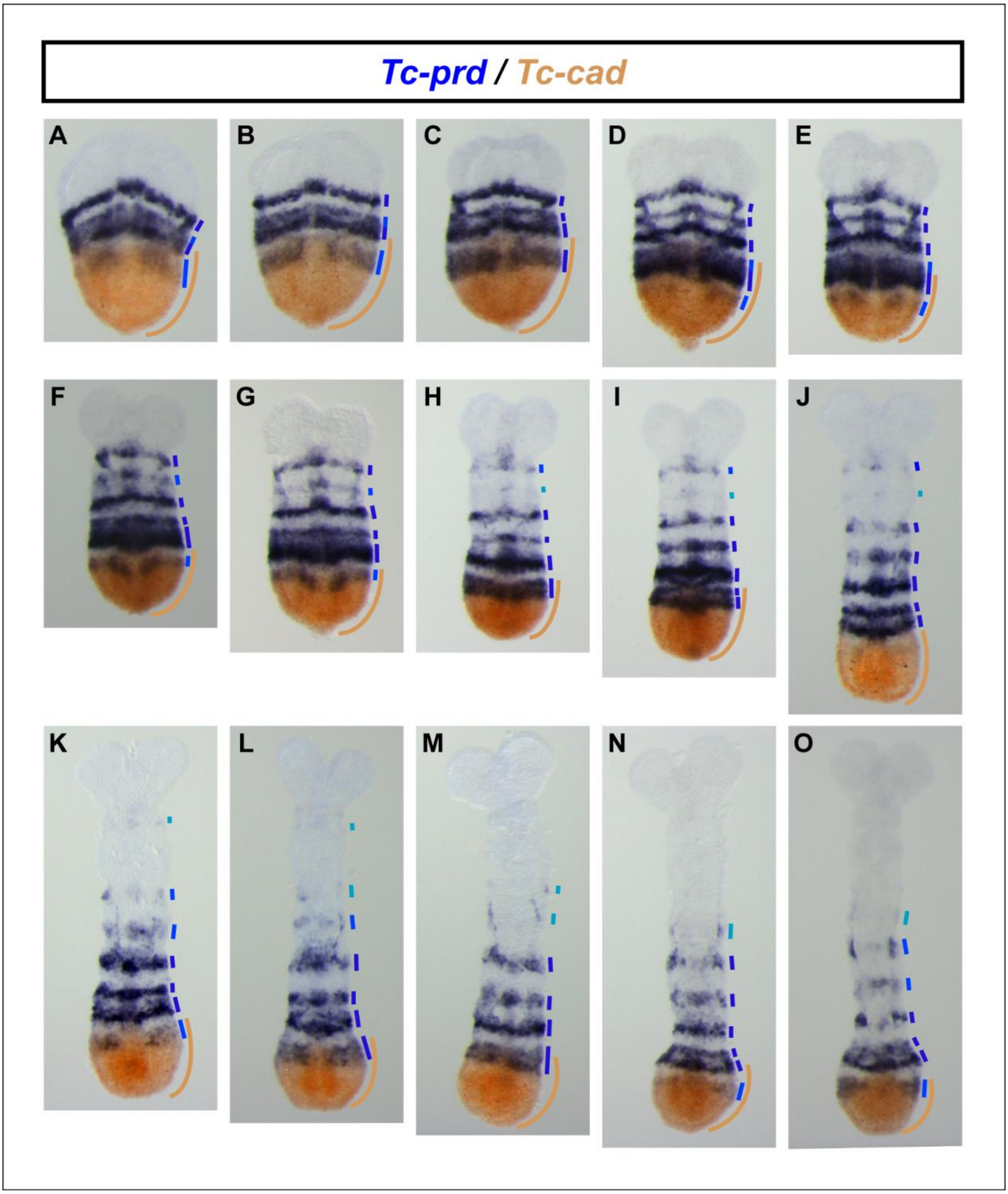

Fig. S7. Expression of *Tc-prd* relative to *Tc-cad* in *Tribolium* germband stage embryos.
(**A-O**) Double in situ hybridization for *Tc-prd* (blue) and *Tc-cad* (brown) in embryos of increasing age from youngest (A) to oldest (O). Colour-coded lines on the right-hand side of the embryos indicate our interpretations of the expression patterns in (A-O). Note how the primary pair-rule stripes of *Tc-prd* first appear and form within the anterior half of the posterior *Tc-cad* domain (see where blue lines overlap brown lines). In contrast, segmental stripes of *Tc-prd* expression resolve by splitting just anterior to the *Tc-cad* domain (see where blue lines lie anterior to the brown line).

Development • Supplementary information

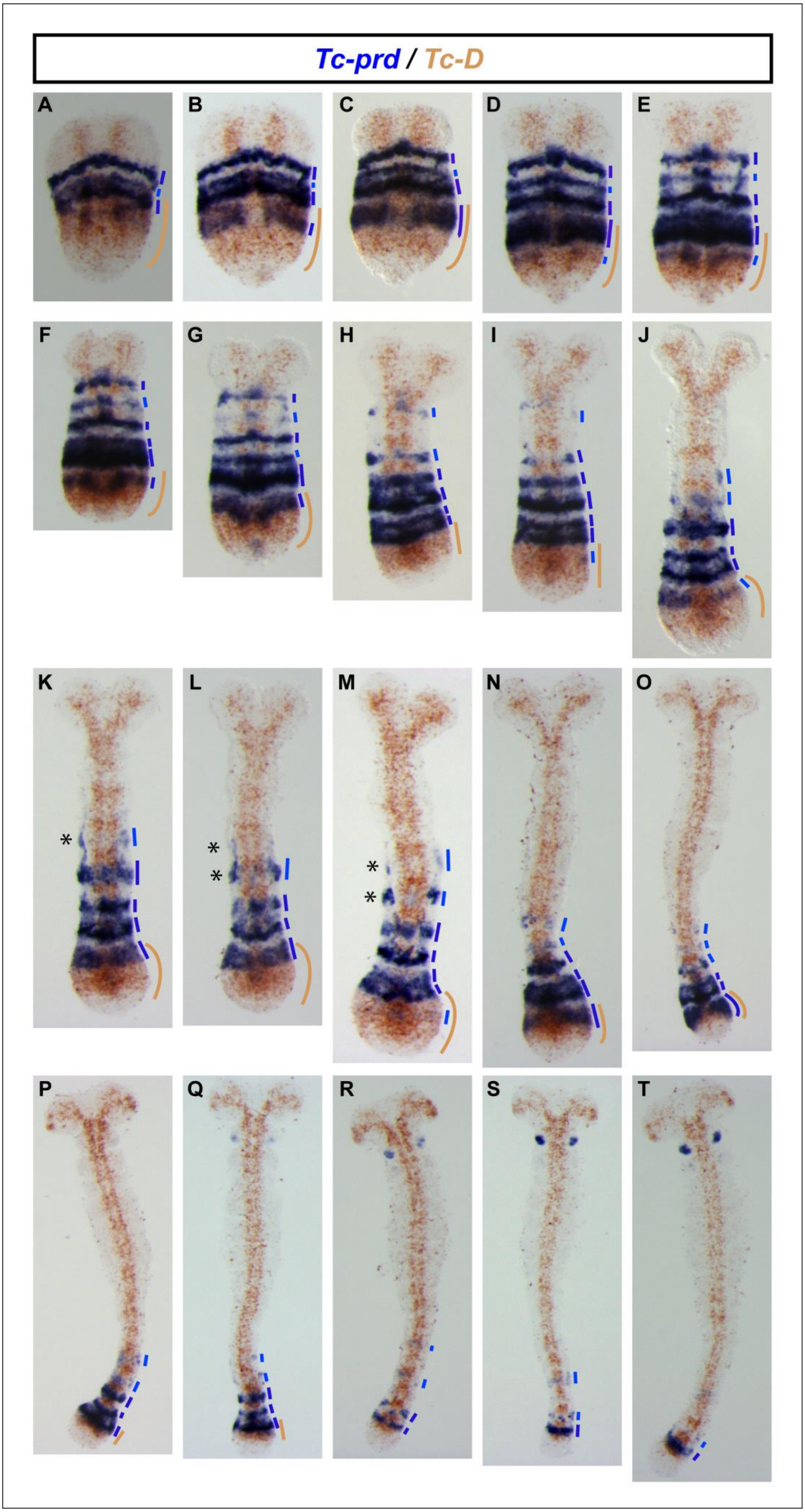

Development • Supplementary information

Fig. S8. Expression of *Tc-prd* relative to *Tc-Dichaete* in *Tribolium* germband stage embryos.
(**A-T**) Double in situ hybridization for *Tc-prd* (blue) and *Tc-Dichaete* (brown) in embryos of increasing age from youngest (A) to oldest (T). Colour-coded lines on the right-hand side of the embryos indicate our interpretations of the expression patterns in (A-T). Note how the primary pair-rule stripes of *Tc-prd* first appear and form within the posterior-most *Tc-Dichaete* domain (see where blue lines overlap brown lines). In contrast, segmental stripes of *Tc-prd* expression resolve by splitting anterior to this domain (see where blue lines lie anterior to the brown line). While dissecting and cleaning the embryos we noted that *Tc-prd* expression remains on stronger and longer in the overlying amnion compared to the underlying ectoderm; this is particularly apparent in panels (K-M), where the *Tc-prd* stained amnion has been ripped away while cleaning the embryo of yolk to reveal ectoderm free from *Tc-prd* expression (asterisks). Amnion-related expression can be seen down the lateral margins of many of the embryos where some amnion cells survived dissection and cleaning.

Development • Supplementary information

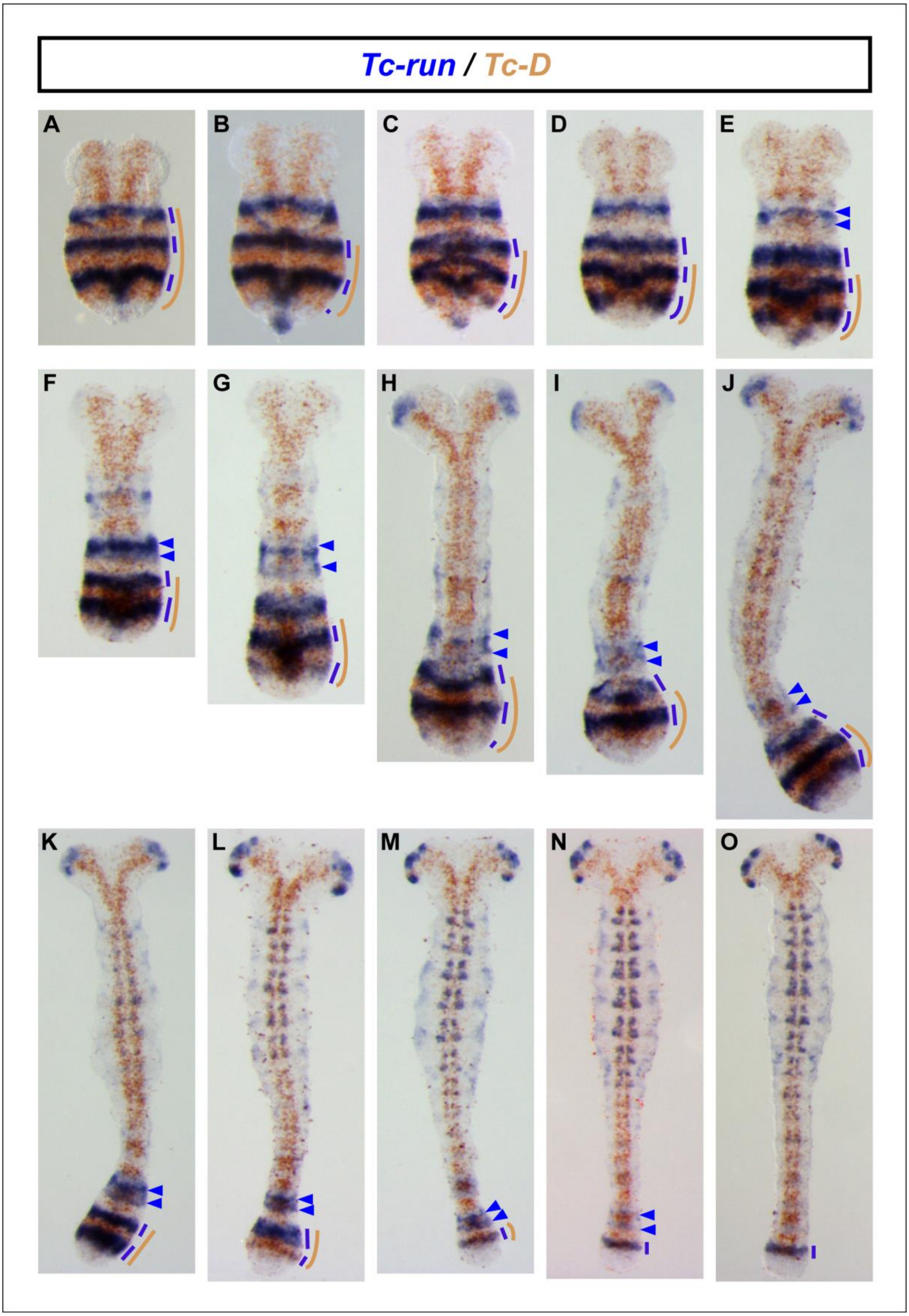

Fig. S9. Expression of *Tc-run* relative to *Tc-Dichaete* in *Tribolium* germband stage embryos.
(**A-O**) Colour-coded lines on the right-hand side of the embryos indicate our interpretations of the expression patterns. Blue arrowheads mark the primary pair-rule stripes that have most recently resolved - or are in the process of resolving – to a segmental periodicity. In some younger embryos (A-H), more than two stripes are apparent due to differences in the timing and/or positioning of this process between the amnion and ectoderm cell layers. Note how *Tc-run* stripe splitting occurs anterior to the posterior-most *Tc-Dichaete* domain (as judged by brown line by side of embryo). Older embryos show additional domains of *Tc-run* expression in the head lobes (H-O) and neuroectoderm (J-O), which were used to help stage the embryos.

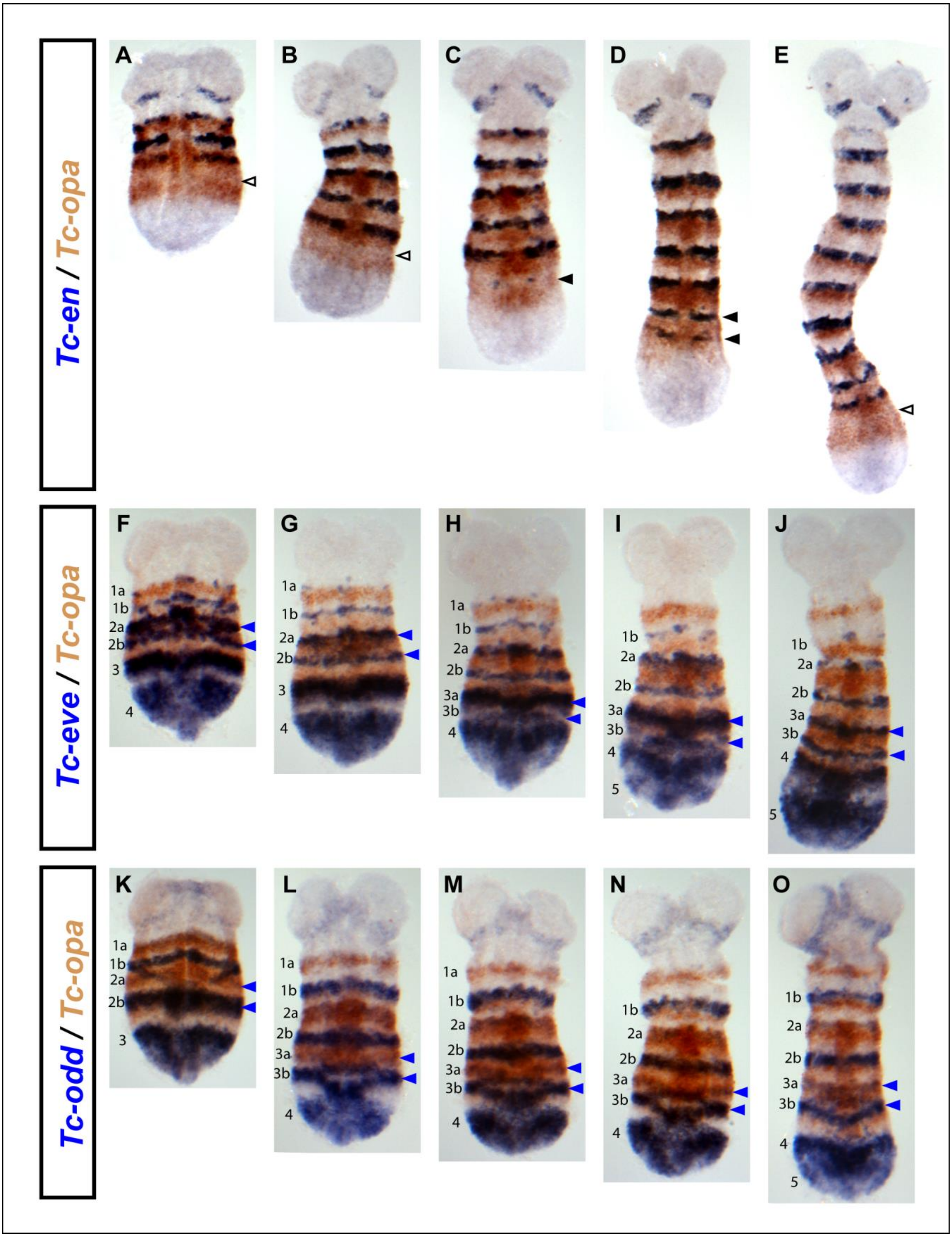

Fig. S10. Expression of *Tc-opa* relative to *Tc-en*, *Tc-eve* and *Tc-odd* in *Tribolium castaneum* germband stage embryos.

(**A-E**) Double in situ hybridization for *Tc-en* (blue) and *Tc-opa* (brown) in embryos of increasing age from left (A) to right (E). Note how the *Tc-en* stripes (solid black arrowheads in C, D) form within the *Tc-opa* domain, but in a stripe-shaped region that is already clearing of *Tc-opa* expression (empty black arrowheads in A, B, E). (**F-J**). As for (A-E), but this time double in situ hybridization for *Tc-eve* (blue) and *Tc-opa* (brown). (**K-O**). As for (A-E), but this time double in situ hybridization for *Tc-odd* (blue) and *Tc-opa* (brown). In (F-O) note how the segmental stripes of *Tc-odd* and *Tc-eve* (labeled a & b) resolve within the *Tc-opa* domain. In (F-O) blue arrowheads mark *Tc-odd* and *Tc-eve* segmental stripes that have most recently resolved, or are in the process of resolving.

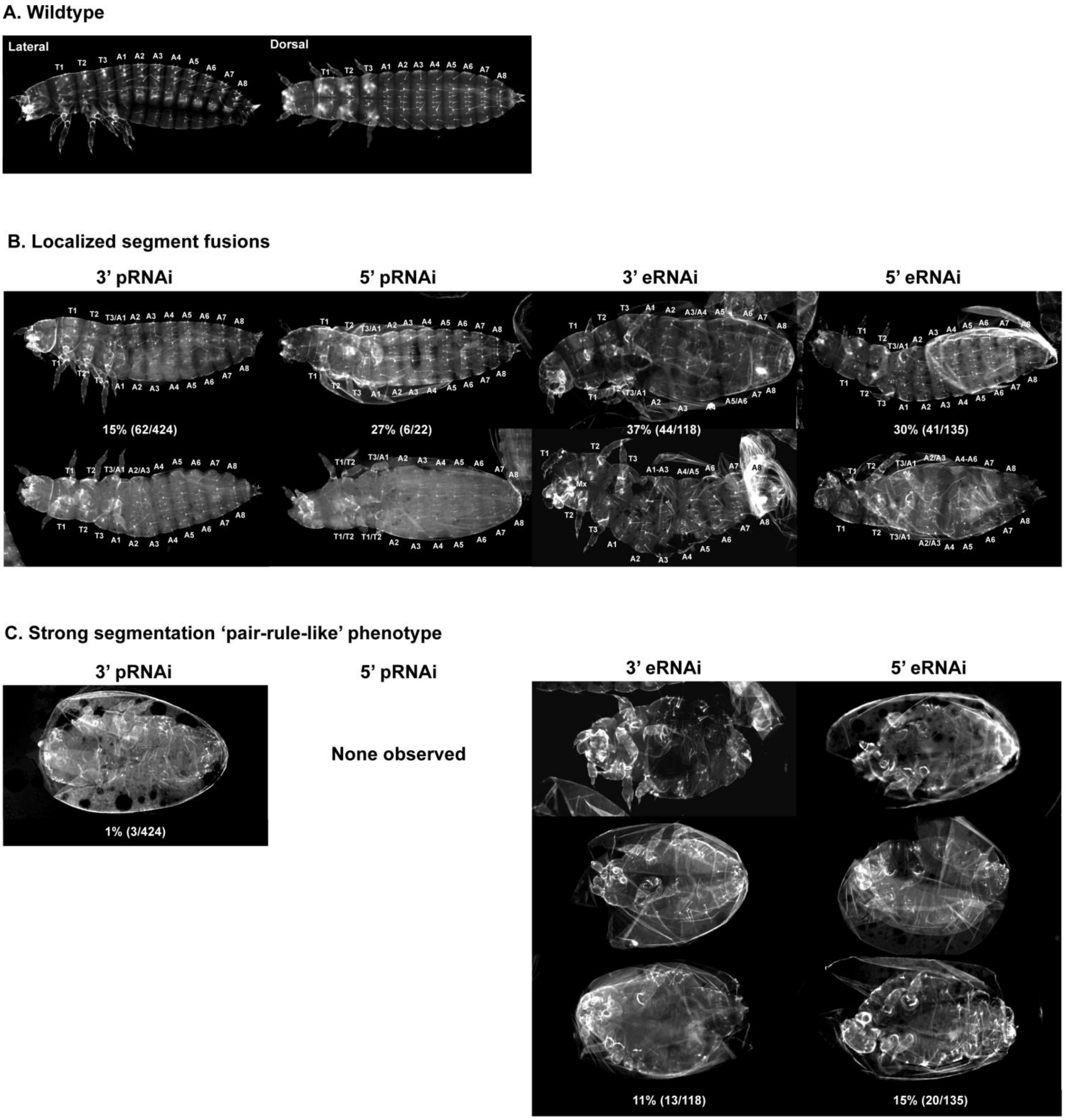

Fig. S11. Similar *Tc-opa* RNAi segmentation phenotypes are observed in distinct RNAi experiments, albeit at differing frequencies.

(A) Wildtype larval cuticles. **(B)** Representative cuticles from 3' parental RNAi (pRNAi), 5' pRNAi, 3' eRNAi and 5' eRNAi experiments, displaying similar local segment fusion phenotypes. The frequency of these phenotypes was between 15% and 37% across the four different RNAi experiments. The relative frequency of cuticles exhibiting local segment fusions, and the number of fused segments per embryo, was higher in eRNAi compared to pRNAi (see Supplementary Tables 1-3 and text for further details). **(C)** Representative cuticles from 3' pRNAi, 3’ eRNAi and 5' eRNAi, experiments displaying similar strong segmentation phenotypes. Less then 1% of cuticles exhibited these phenotypes in 3' pRNAi, and none were observed in 5' pRNAi, whereas their number and frequency was higher following 3' & 5' eRNAi (11-15%). Three representative cuticles are shown for each eRNAi experiment to illustrate the consistent ‘pair-rule-like’ appearance of these phenotypic cuticles; i.e. T1 & T2 legs fused, mandibular and labial appendages often lost, and only 4 abdominal segments obvious.

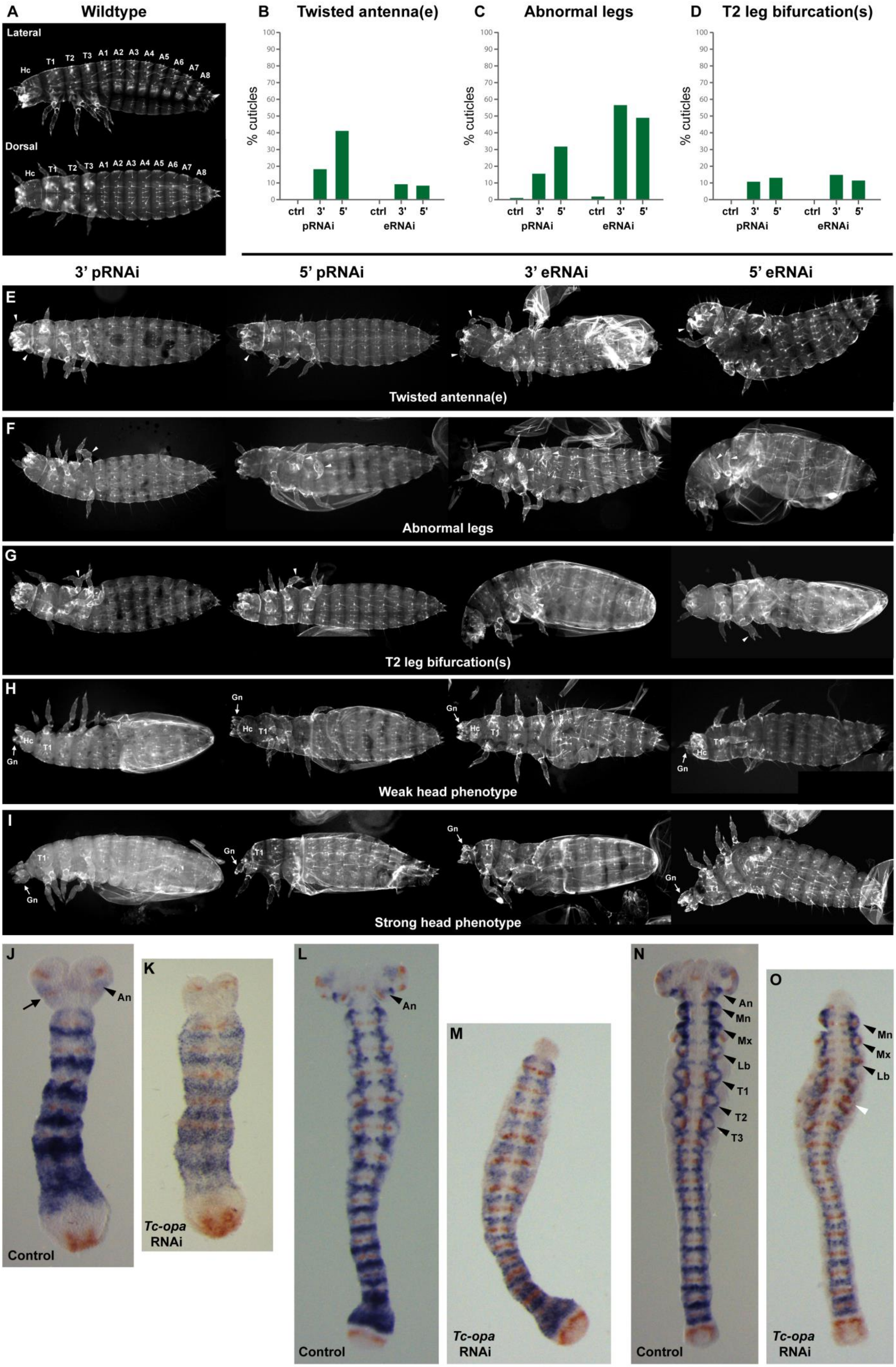

Development • Supplementary information

Fig S12. Similar *Tc-opa* RNAi appendage and head phenotypes are observed in distinct RNAi experiments, with associated defects found in *Tc-opa* pRNAi germband embryos.
(**A**) Wildtype larval cuticles. (**B-D**) The relative frequency of twisted antenna(e), abnormal leg(s) and T2 leg bifurcation(s) larval phenotypes observed in the 3' pRNAi, 5' pRNAi, 3' eRNAi and 5' eRNAi experiments, compared with sham injection controls. NB. An equivalent graph for head phenotypes is shown in Fig. 7. Refer to Tables S1-3 for exact details of the relative frequency of these phenotypes across the RNAi experiments and their controls. (**E**) Representative cuticles from each RNAi experiment exhibiting either one or two antennae that are abnormally twisted backwards (white arrowheads). (**F**) Representative cuticles from each RNAi experiment showing abnormalities in leg development (white arrowheads); note that these deformities included one or more of the following: twisted leg, short leg (absorbed into body wall), fused leg segments, bifurcated leg. (**G**) Representative cuticles from each RNAi experiment exhibiting asymmetric T2 leg bifurcations (white arrowheads); note that the proximodistal position of these bifurcations varied (from femur to claw). This phenotype represents a common subclass within the 'abnormal leg(s)' class of phenotype. (**H**) Representative cuticles from each RNAi experiment with weak head phenotypes, defined as a reduced head capsule (Hc) and/or labrum, judged in relation to the size of the prothoracic segment (T1). (**I**) Representative cuticles from each RNAi experiment with strong head phenotypes, defined as a complete absence of the head capsule (Hc), labrum and antennae, with only gnathal appendages (Gn) remaining. (**J-O**) Germband stage embryos from *Tc-opa* pRNAi females (K, M, O) compared to stage-matched embryos (using *Tc-wg* expression; red) from sham-injected control females (J, L, N). *Tc-opa* pRNAi germband embryos exhibit abnormalities that correlate with the antennal and head larval cuticle phenotypes: (**i**) Reduced (K) or missing (M, O) head lobes, likely reflecting the weak (H) and strong (I) head cuticle phenotypes respectively. (**ii**) Missing antennal *Tc-opa* (black arrowhead in J) & *Tc-wg* (black arrow in J) expression domains (compare J to K), possibly linked to the twisted antenna(e) phenotype (E). (NB. We suspect that disruption of the early blastoderm wedge shape domain shown in Fig. 8, which covers the future antennal segment, is linked to the broad head phenotypes (H, I), whereas disruption of the later domains of *Tc-opa* expression within the antennal segment is responsible for twisted antenna(e)). (**iii**) Reduced *Tc-opa* expression at the base of, and/or surrounding, developing appendages (black arrowheads in N; compare to the stage-matched *Tc-opa* RNAi embryo in O). Note that *Tc-opa* expression within and/or surrounding some gnathal appendages (i.e. mandibles; Mn & maxillae; Mx in N) is much stronger than that seen in/around leg appendages, and remains relatively strong in RNAi embryos (O), perhaps explaining why gnathal appendages were refractory to our *Tc-opa* RNAi. (**iv**) A patch of ectopic *Tc-wg* expression on the left side of the T2 segment (white arrowhead in O), is likely associated with the T2 leg bifurcations we observe in cuticles (G); note that the knockdown of *Tc-opa* seems quite efficient in the T2 segment (O), perhaps resulting in the derepression of *Tc-wg* (see Discussion).

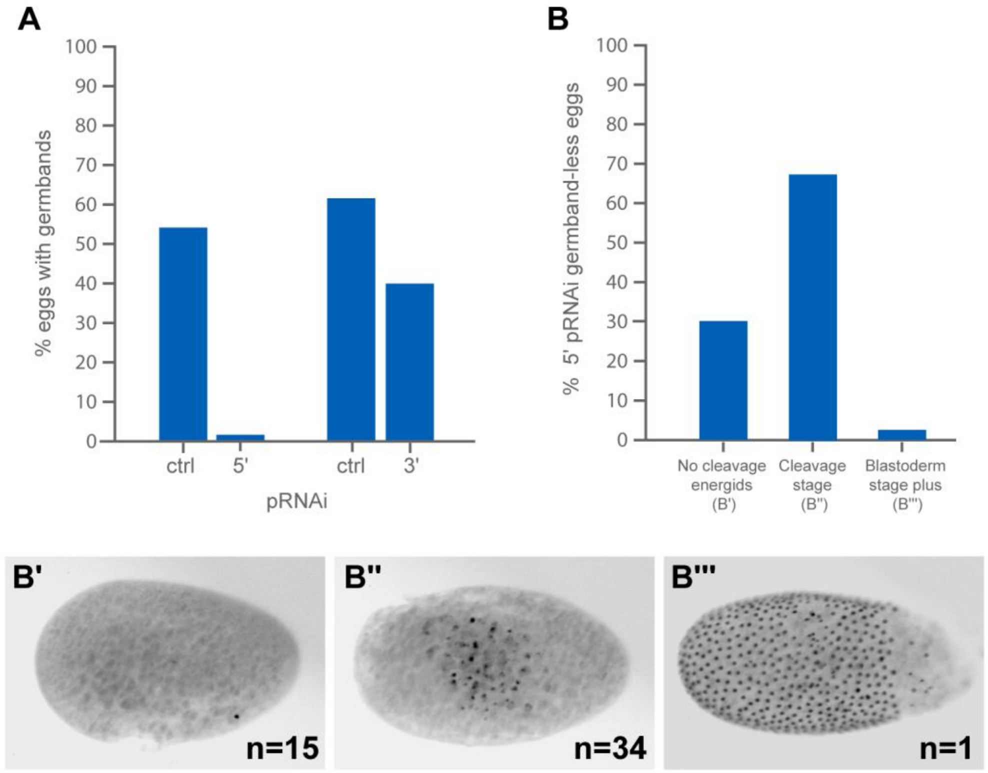

Fig. S13. The *Tc-opa* RNAi blastoderm phenotype.
(**A**) The percentage of eggs that had reached the germband stage in early 48-hour (30°C) egg collections taken from 3' and 5' *Tc-opa* parental RNAi (pRNAi) females and their parallel control (buffer) injected females. Both 3' and 5' *Tc-opa* pRNAi results in a drop in the percentage of germband stage embryos relative to controls, however this reduction is much more dramatic with 5' *Tc-opa* pRNAi. (**B**) A random sample of 50 germband-less eggs was taken from the same 5' *Tc-opa* pRNAi egg collection as shown in (A) and stained with DAPI. Despite being up to 48-hours old, only one egg (2%) had formed a blastoderm; this egg is shown in panel (B'''). The majority of eggs (68%) exhibited cleavage nuclei within the yolk, suggesting that in most of these eggs embryogenesis had started, but development had stalled prior to blastoderm stage (one of these eggs is shown in B''). The remaining 30% of eggs showed no sign of cleavage nuclei (although a polar body nuclei was clearly present in some cases; example shown in B'). However, it cannot be ruled out that some of these eggs possessed early cleavage nuclei undetected deeper within the yolk.

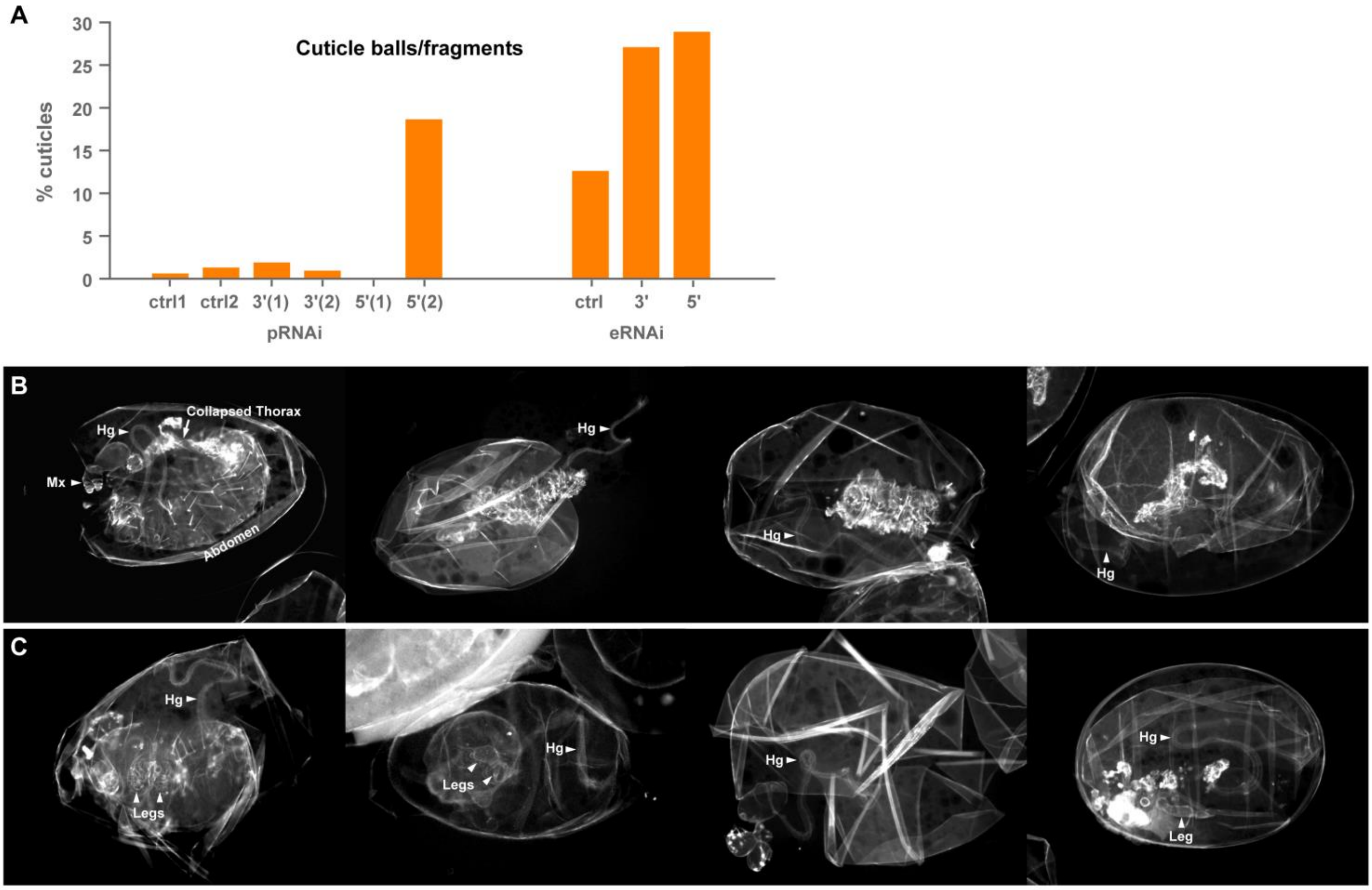

Fig. S14. Increased frequency of cuticle ball and cuticle fragment phenotypes following *Tc-opa* RNAi. (**A**) The percentage of cuticles scored as 'cuticle balls' and/or 'cuticle fragments' following pRNAi or eRNAi and associated parallel injection controls. Cuticle balls/fragments were observed in higher numbers in our second 5' pRNAi experiment (see discussion associated with Table S1), and in 3' and 5' eRNAi experiments, when compared to injection controls. (**B-C**) Examples of eRNAi eggs containing cuticle balls and/or cuticle fragments arranged in two highly speculative phenotypic series. Note that in each of the eight images a fully developed hindgut (Hg) is present, suggesting that this aspect of development proceeded as normal. The speculative phenotypic series in row (B) begins on the left with a cuticle that would have been classified as a strong head phenotype (note the lone pair of maxillae and almost complete abdomen) had its thoracic and/or anterior abdominal segments(?) not collapsed and shriveled up into a bristle lined cylinder. Numerous cuticles assigned to this phenotypic class were bristle-lined cylinder-shaped cuticles (with an absence of other discernible features); increasingly severe examples are shown along row (B). In contrast, the speculative phenotypic series in row (C) begins on the left with a large cuticle ball that could be interpreted as an extreme segmentation phenotype, with gnathal appendages perhaps present but indecipherable, evidence of only extremely short legs and less than 4 clear abdominal segments. Numerous cuticles assigned to this phenotypic class were smaller cuticle balls, attached to – or alongside – a fully developed hindgut, whereas other eggs exhibited cuticles that appeared to have broken up, with some recognizable structures remaining (e.g. a fully developed leg); examples of these are arranged in order of increasing severity along row (C). These cuticles are almost impossible to interpret, since no two are entirely alike, and examples are also observed following embryonic control injections. However, given their increased relative frequency in 3' and 5' *Tc-opa* eRNAi compared to controls, and their observation in a 5' pRNAi experiment (i.e. arguing against injection artifacts being solely responsible), it is possible that at least some of these cuticles are the direct, or indirect, result of strong *Tc-opa* RNAi knockdowns, and represent extreme head and/or segmentation phenotypes. This may explain why in the *Tc-opa* eRNAi experiments only 10-15% of eggs exhibit clear and interpretable pair-rule-like phenotypes.

Supplementary Table 1

The percentage of cuticles that exhibited each class of egg or cuticle phenotype in each of the parental RNAi experiments and their corresponding parallel injection controls. Table includes total number of eggs or cuticles scored.

	**Parental RNAi**						
	Experiment 1				Experiment 2		
	3'		5'		3'	5'	Cont.
	RNAi	Cont.	RNAi	Cont.			
**Eggs examined (n)**	**578**	**645**	**392**	**480**	**300**	**300**	**284**
Empty eggs (%)	26.6	16.1	94.1	71.7	22.7	85.7	19.4
Wildtype cuticles (%)	37.9	81.9	1.0	23.3	27.3	2.0	76.1
Phenotypic cuticles (%)	35.5	2.0	4.6	0.2	50.0	12.3	4.6
Not scorable (%)	-	-	0.3	4.8	-	-	-
**Cuticles scored (n)**	**424**	**541**	**22**	**113**	**232**	**43**	**229**
Wildtype cuticles (%)	51.7	97.6	18.2	99.1	35.3	14.0	94.3
Phenotypic cuticles (%)	48.3	2.4	81.8	0.9	64.7	86.0	5.7
Antennae abnormal (lost, reduced, twisted, bifurcated) (%)	29.0	0.0	77.3	0.9	44.8	67.4	0.0
Antennae twisted backwards (%)	*18.4*	*0.0*	*40.9*	*0.0*	*25.0*	*37.2*	*0.0*
Leg(s) abnormal (twisted, bifurcated, short, fused segments) (%)	16.0	0.6	31.8	0.0	30.6	30.2	0.9
At least one T2 leg bifurcated (branching position varies) (%)	*10.6*	*0.0*	*13.6*	*0.0*	*18.5*	*14.0*	*0.0*
Anterior head (head capsule and/or labrum) reduced (%)	16.0	0.0	45.5	0.0	22.0	34.9	0.4
Weak (head capsule and/or labrum present, but reduced) (%)	14.2	0.0	27.3	0.0	15.5	14.0	0.0
Strong (missing, usually only gnathal appendages remain) (%)	1.9	0.0	18.2	0.0	6.5	20.9	0.4
Total segment fusion phenotypes (%)	15.3	0.0	27.3	0.0	21.6	11.6	0.9
Total cuticles with local segment fusions (%)	14.6	0.0	27.3	0.0	21.1	11.6	0.9
Fusion of T3/A1 only (%)	*11.1*	*0.0*	*18.2*	*0.0*	*16.4*	*7.0*	*0.0*
Total cuticles with T3/A1 fusions (%)	*13.2*	*0.0*	*22.7*	*0.0*	*17.7*	*9.3*	*0.0*
Local segment fusions in segment(s) other than T3/A1 (%)	*3.5*	*0.0*	*9.1*	*0.0*	*4.7*	*4.7*	*0.9*
Strong ‘pair-rule’ phenotype (all segments affected) (%)	0.7	0.0	0.0	0.0	0.4	0.0	0.0
Cuticle ball and/or cuticle fragments (%)	1.9	0.6	0.0	0.0	0.9	18.6	1.3
Miscellaneous abnormalities	2.1	1.3	9.1	0.0	4.3	7.0	4.4

In the first round of pRNAi experiments 3' and 5' dsRNA was injected into adult females on different days, each time alongside parallel injection controls, such that there is a control group associated with each dsRNA fragment. The same population (box) of animals was used, and subsequent egg collections were made at the same times in relation to the day of injection. In the second round of pRNAi experiments, 3' and 5' dsRNA was injected on the same day, alongside one set of injection controls. In the first 5' pRNAi experiment, cuticle preparations were made before all eggs would have had the opportunity to secrete cuticles. It is notable that the control eggs possessed a significant number of embryos that were in the process of secreting cuticle (23/480), and therefore “Not scorable” as wildtype or phenotypic cuticles (fifth line of table). In contrast, 5' pRNAi eggs possessed very few developing embryos (1/392), consistent with the higher level of empty eggs in this experiment. The second round of pRNAi experiments was therefore carried out partly to gain a more accurate measure of the frequency of empty eggs, but also acted as an experimental repeat. In order to gain a more accurate comparison between the frequencies of empty eggs in pRNAi

Development • Supplementary information

vs. eRNAi experiments, in the second pRNAi experiments eggs from injected females were lightly bleached and lined up on slides, as they would be for embryonic injection.

We note that the second set of 3' and 5' pRNAi experiments appear to have resulted in stronger knockdowns. Importantly, the same classes of phenotype were observed across all pRNAi experiments. However, the frequency of phenotypes, and/or the frequency of stronger phenotypes relative to weaker ones, was generally higher in the second round of pRNAi experiments. Of particular note is the higher number of cuticle balls/fragments observed in the second 5' pRNAi experiment. There are a number of potential explanations for this, none mutually excusive: (i) 5' pRNAi eggs (i.e. stronger knockdowns) were more sensitive to the mechanical manipulation associated with lining eggs up on slides; this might also explain the high number of cuticle balls/fragments seen in eRNAi experiments. (ii) Cuticle ball/fragment phenotypes represent a stronger knockdown than strong head phenotypes (see Fig. S14). This is supported by the observation that although the frequency of head phenotypes was lower overall in the second 5' pRNAi experiment, there was a higher proportion of strong head phenotypes (60% vs. 40%). (iii) The lower number of cuticles obtained and scored for the 5' pRNAi experiments (e.g. 43 compared to 232 for 3' pRNAi), means that the data are more sensitive to random variations.

Supplementary Table 2

The percentage of cuticles that exhibited each class of egg or cuticle phenotype in each of the embryonic RNAi experiments and their corresponding parallel injection controls. Table includes total number of eggs or cuticles scored.

	Embryonic RNAi		
	3'	5'	Controls
**Eggs examined (n)**	**198**	**252**	**198**
Empty eggs (%)	40.4	46.4	27.8
Wildtype cuticles (%)	3.5	7.5	55.1
Phenotypic cuticles (%)	56.1	46.0	17.2
**Cuticles scored (n)**	**118**	**135**	**143**
Wildtype cuticles (%)	5.9	14.1	76.2
Phenotypic cuticles (%)	94.1	85.9	23.8
Antennae abnormal (lost, reduced, twisted, bifurcated) (%)	32.2	34.1	2.1
Antennae twisted backwards (%)	*9.3*	*8.1*	0.0
Leg(s) abnormal (twisted, bifurcated, short, fused segments) (%)	56.8	48.9	1.4
At least one T2 leg bifurcated (branching position varies) (%)	*15.3*	*11.1*	0.0
Anterior head (head capsule and/or labrum) reduced (%)	31.4	32.6	1.4
Weak (head capsule and/or labrum present, but reduced) (%)	17.8	18.5	0.0
Strong (missing, usually only gnathal appendages remain) (%)	13.6	14.1	1.4
Total segment fusion phenotypes (%)	48.3	45.2	2.8
Total cuticles with local segment fusions (%)	37.3	30.4	2.8
Fusion of T3/A1 <u>only</u> (%)	*4.2*	*5.9*	0.0
Total cuticles with T3/A1 fusions (%)	*19.5*	*20.7*	*0.0*
Local segment fusions in segment(s) other than T3/A1 (%)	*33.1*	*24.4*	*2.8*
Strong 'pair-rule' phenotype (all segments affected) (%)	11.0	14.8	*0.0*
Cuticle ball and/or cuticle fragments (%)	27.1	28.9	12.6
Miscellaneous abnormalities	4.2	7.4	8.4

Development • Supplementary information

Supplementary Table 3

The percentage of cuticles that exhibited each class of egg or cuticle phenotype in each of the parental and embryonic RNAi experiments. Table includes total number of eggs or cuticles scored.

	Parental RNAi				Embryonic RNAi	
	Experiment 1		Experiment 2			
	3'	5'	3'	5'	3'	5'
**Eggs examined (n)**	**578**	**392**	**300**	**300**	**198**	**252**
Empty eggs (%)	26.6	94.1	22.7	85.7	40.4	46.4
Wildtype cuticles (%)	37.9	1.0	27.3	2.0	3.5	7.5
Phenotypic cuticles (%)	35.5	4.6	50.0	12.3	56.1	46.0
Not scorable (%)	-	0.3	-	-	-	-
**Cuticles scored (n)**	**424**	**22**	**232**	**43**	**118**	**135**
Wildtype cuticles (%)	51.7	18.2	35.3	14.0	5.9	14.1
Phenotypic cuticles (%)	48.3	81.8	64.7	86.0	94.1	85.9
Antennae abnormal (lost, reduced, twisted, bifurcated) (%)	29.0	77.3	44.8	67.4	32.2	34.1
Antennae twisted backwards (%)	*18.4*	*40.9*	*25.0*	*37.2*	*9.3*	*8.1*
Leg(s) abnormal (twisted, bifurcated, short, fused segments) (%)	16.0	31.8	30.6	30.2	56.8	48.9
At least one T2 leg bifurcated (branching position varies) (%)	*10.6*	*13.6*	*18.5*	*14.0*	*15.3*	*11.1*
Anterior head (head capsule and/or labrum) reduced (%)	16.0	45.5	22.0	34.9	31.4	32.6
Weak (head capsule and/or labrum present, but reduced) (%)	14.2	27.3	15.5	14.0	17.8	18.5
Strong (missing, usually only gnathal appendages remain) (%)	1.9	18.2	6.5	20.9	13.6	14.1
Total segment fusion phenotypes (%)	15.3	27.3	21.6	11.6	48.3	45.2
Total cuticles with local segment fusions (%)	14.6	27.3	21.1	11.6	37.3	30.4
Fusion of T3/A1 only (%)	*11.1*	*18.2*	*16.4*	*7.0*	*4.2*	*5.9*
Total cuticles with T3/A1 fusions (%)	*13.2*	*22.7*	*17.7*	*9.3*	*19.5*	*20.7*
Local segment fusions in segment(s) other than T3/A1 (%)	*3.5*	*9.1*	*4.7*	*4.7*	*33.1*	*24.4*
Strong 'pair-rule' phenotype (all segments affected) (%)	0.7	0.0	0.4	0.0	11.0	14.8
Cuticle ball and/or cuticle fragments (%)	1.9	0.0	0.9	18.6	27.1	28.9
Miscellaneous abnormalities	2.1	9.1	4.3	7.0	4.2	7.4
